# Ministerial Autonomy, Parliamentary Scrutiny and Government Reform Output in Parliamentary Democracies

**DOI:** 10.1177/00104140211024312

**Published:** 2021-07-02

**Authors:** Hanna Bäck, Wolfgang C. Müller, Mariyana Angelova, Daniel Strobl

**Affiliations:** 1 225311 Lund University, Sweden; 2 31290 University of Vienna, Austria; 3 Central European University, Austria

**Keywords:** cabinets, ministerial government, economic and social policy, coalition governance, parliamentary scrutiny

## Abstract

One of the most important decisions coalition partners make when forming a government is the division of ministries. Ministerial portfolios provide the party in charge with considerable informational and agenda-setting advantages, which parties can use to shape policies according to their preferences. Oversight mechanisms in parliaments play a central role in mitigating ministerial policy discretion, allowing coalition partners to control each other even though power has been delegated to individual ministers. However, we know relatively little about how such mechanisms influence the agenda-setting and gatekeeping powers of ministers and how much influence minister parties have on policy output relative to the government as a whole in different institutional settings. We fill this gap by analyzing original data on over 2000 *important* social and economic policy reform measures adopted in nine Western European countries over 20 years, based on a coding of more than 1200 country reports issued by the *Economist Intelligence Unit* and the *Organisation for Economic Co-operation and Development (OECD)*. We find that parliaments with strong oversight powers constrain the agenda-setting capacity of minister parties but have limited impact on their gatekeeping capacity. Our findings have important implications for our understanding of policy-making and democratic accountability.

## Introduction

How important are ministerial portfolios for policy influence in coalition settings? During the government formation process in Germany in 2017, Angela Merkel, the leader of the *formateur* party – Christian Democratic Union (CDU) – was accused of making ‘painful concessions to form new government’ (Spiegel, 2018, see also Nasr, 2018). The CDU agreed to delegate control over the ‘critical Finance Ministry, which will be central to any reforms of the European common currency’ (Spiegel, 2018) to its coalition partner the Social Democratic Party (SPD). Merkel put these accusations in perspective by pointing to the mutual decision making process and the veto power of governing parties in coalitions: ‘I want to say that we (the conservatives) have also approved the policies (in the agreement) and the finance minister cannot simply do as he likes’ (Nasr, 2018). However, given the varying dynamics of the decision making process in coalitions in different institutional settings, it remains unclear whose preferences eventually shape policy-making in coalition governments and to what extent. In particular, we still know little about whether and when the coalition as a whole, as suggested by Merkel, or the party in charge of a ministry, as implied by the media in the above example, shapes policy output.

Understanding who shapes government policy is critical for democratic accountability and representation. The vast majority of parliamentary governments consist of multiple parties (see e.g. Strøm et al., 2008). Every multiparty government in the world faces the critical decision of dividing ministerial portfolios between the coalition parties. In turn, this means that most coalition parties will find themselves in the position of needing to relinquish control over desired ministries to their coalition partners. What are the actual policy costs of losing a desired ministry? We know that ministries are essential for policy-making. In particular, ministers can influence governmental policy output by deciding (1) which policy proposals to put forth to the cabinet; (2) the initial form and substance of such proposals (positive agenda power); and (3) which policy issues to leave out of the cabinet agenda (gatekeeping) (Laver & Shepsle, 1996; Martin, 2004). Given this privileged position, Laver and Shepsle (1990, 1996) argue that ministers and their parties are akin to ‘policy dictators’ within their jurisdictions.

However, scholars have disputed the autonomy of ministers in their assigned policy area and have shown that coalition partners use various monitoring and oversight mechanisms to reduce the informational advantage of ministers and constrain their policy influence (Carroll & Cox, 2012; Kim & Loewenberg, 2005; Lipsmeyer & Pierce, 2011; Fernandes et al., 2016; Martin, 2004; Martin and Vanberg, 2004; Martin and Vanberg, 2004, 2005, 2011; Thies, 2001). Nevertheless, while we know that such control mechanisms are widely used, we do not have a good understanding of how successful they are (for an exception, see e.g. Martin & Vanberg, 2020) and how important ministerial portfolios are for party influence on government policy output.

We investigate this question by analyzing original data on over 2000 *important* social and economic *reform measures* passed by coalition governments in nine Western European countries between 1985 and 2005, which we collected by coding more than 1200 country reports by the *Economist Intelligence Unit* and the *Organisation for Economic Co-operation and Development (OECD)*. While previous studies on ministerial policy-making predominantly focus on one policy issue (e.g. unemployment generosity) (see Alexiadou, 2015; Bäck et al., 2015; Becher, 2010; Martin & Vanberg, 2020), we provide a broader focus and analyze reforms in the social and economic policy domains, covering 11 policy areas.

Our findings indicate that parliamentary policing instruments constrain the agenda-setting power of ministers and thus limit their impact on governmental policy output. However, such instruments have little effect on the gatekeeping power of ministers – to decide over which policies to propose to the cabinet. Consequently, the ability of coalition partners to correct ministerial decisions and actively structure policy output in accordance with the overall coalition position at the parliamentary stage remains relatively limited. These findings have important implications for our understanding of coalition policy-making, the role of various oversight mechanisms, and more broadly for democratic accountability and representation.

## Policy-Making in Coalition Governments

### Delegation Problem in Coalition Governments and Ministerial Policy-Making Powers

Policy-making in multiparty governments is characterized by several challenges. Coalition partners have differing preferences and compete at elections separately, but they govern together and need to agree on a common policy position to pass legislation. Thus, each coalition party has strong incentives to advance its own policy interests and satisfy its own voters.

The *inter*-party delegation process in coalition cabinets exacerbates the problem of conflicting incentives (Martin and Vanberg, 2004, 2011, 2014). The need to divide labour and acquire policy expertise to deal with complex issues and draft feasible legislation has led to the departmentalization of the cabinet, where ministries specialize in the policy area(s) under their purview, and have exclusive power to make proposals to the cabinet. As a result of the jurisdictional system, cabinets are forced to delegate considerable authority to individual ministers. Through the allocation of ministerial portfolios, each party in charge of a ministry gains access to vast resources and technical policy expertise (Laver & Shepsle, 1996). This provides ministers with valuable information on what is feasible in a given policy area at a given point of time and what will be the outcome of particular policy decisions (Martin & Vanberg, 2004), which implies that ministers are the only ones who have the resources and technical expertise to develop a viable proposal. In essence, the delegation process in cabinet grants ministers and their departments extensive agenda-setting and gatekeeping powers.

Given differing policy preferences between coalition partners, ministers and their parties have strong incentives to use this informational advantage to advance their own interests in the policy-making process. Specifically, they can choose which policy initiatives to feed into the cabinet and how these are shaped (positive agenda-setting). Alternatively, they can decide which policies to delay or suppress (negative agenda-setting or gatekeeping) (Martin, 2004). In the face of ‘political uncertainty’, ministers can draft a bill, which deviates from the agreed-upon position in the coalition, and present it as the ‘best’ feasible policy given the complexities of the current situation (Martin & Vanberg, 2004). Alternatively, they can shelve the policy issue for the time being, or indefinitely, referring to policy complexities, or to other policy issues being of higher priority. Due to time constraints and lack of access to policy-specific information and civil servant specialists in departments under the control of their coalition partners, parties in government have limited ability to shape the drafting process of bills in the ministries controlled by their coalition partners (Laver & Shepsle, 1996).

The delegation problems in coalition governments and the informational advantage of ministers are undisputed in the literature. Scholars, however, disagree on how partners deal with these challenges. The seminal work by Laver and Shepsle (1990, 1996) on portfolio allocation suggests that government partners accept ministerial autonomy and thus abdicate any influence in the policy areas controlled by their partners (see also Austen-Smith & Banks, 1990). This ‘ministerial government’ perspective suggests that the preferences of minister parties will shape government policy output in the policy areas under their purview.

**Figure 1.**
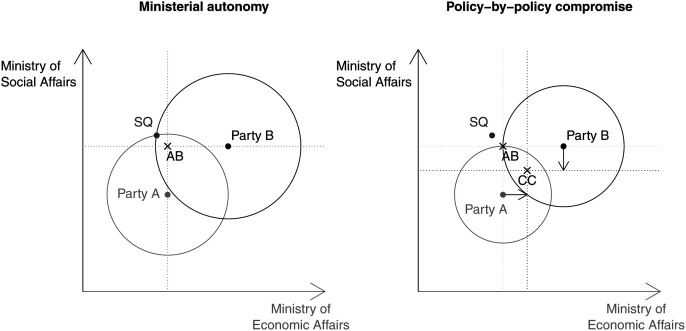
Ministerial autonomy versus policy-by-policy compromise.

This situation is illustrated in Figure 1 (left panel) (see also Thies, 2001; Martin & Vanberg, 2020). For illustration purposes, take as an example a coalition between two parties, A and B. Their ideal positions on a two-dimensional space, social and economy policy, are indicated as points A and point B, and the inherited status quo is indicated by the point SQ. If we draw indifference curves through the status quo, the intersection of both circles covers all positions where both parties are better off as compared to the status quo position. In this hypothetical coalition, party A controls the ministry of economic affairs and party B the ministry of social affairs. Accordingly, under the ministerial autonomy scenario, the final government position will lie at point AB, where each party gets its ideal position in the policy area it controls.

Thies (2001) shows that while the ‘ministerial government’ perspective is incentive-compatible, because each party gets its ideal position in the policy area under its ministerial control, the final policy outcome is Pareto inferior. Under many circumstances, coalition partners prefer a policy-by-policy compromise in all policy areas (Thies, 2001; Martin & Vanberg, 2004). This is illustrated in the right-hand panel of Figure 1, which depicts the same coalition government, with the difference that the indifference curves go through the government position AB, where parties get their ideal positions in the policy areas under their ministerial purview. Once the indifference curves go through the point AB, we can see that there are many positions, which would make both parties better off as compared to the ministerial autonomy outcome at AB (see the overlap of the indifference curves of both parties). One such point is the point CC (policy-by-policy compromise). Both parties prefer that the government policy output ends at CC rather than at AB. This, in turn, requires the credible commitment of all coalition partners to the agreed policy compromise, meaning that each government party is expected to introduce the compromise position in the ministries it controls.

**Figure 2.**
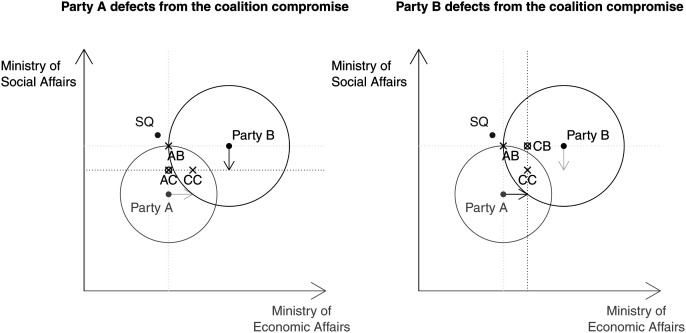
Ministerial drift.

Although coalition partners would benefit from a policy-by-policy compromise, they are not likely to commit to a compromised policy in their jurisdictions. Given differing policy preferences and informational asymmetries, they have the incentives and the opportunity to defect from the agreed-upon policy compromise (Thies, 2001; Martin & Vanberg, 2005). Figure 2 shows the final government policy outcome if only one party commits to the agreed-upon policy-by-policy compromise and the other partner defects. The left-hand panel of Figure 2 depicts a scenario where party B commits to the compromise and introduces the compromised policy in the social affairs jurisdiction which it controls, while party A only commits to do so, but eventually introduces its ideal position in the economic policy area. Ministerial informational advantage enables Party A to get through with it. Given policy complexity and uncertainty, as the responsible minister party, party A is the only one who has access to sufficient policy expertise and resources to evaluate the current policy situation and the effectiveness of policy instruments. Party A can use its informational advantage to credibly argue that, given the new circumstances, the previously agreed compromise in the economic policy area is not feasible. Under such constellations, the government position would end up at point AC, at which A gets its ideal point in the economic policy area, and B introduces the agreed-upon compromise in the social policy area. The government position at AC is outside of the indifference curve of party B and is thus worse than the ministerial autonomy outcome at AB for party B. The reverse is true if party A commits to the agreed policy-by-policy compromise, while party B defects, ending up at point CB (see the right panel in Figure 2). Essentially, the worst-case scenario for any party in the coalition is when it makes policy concessions, while its partner(s) does not.

Coalition partners can solve this commitment problem through various monitoring and control mechanisms in cabinet and parliament, which provide coalition partners with policy-specific information and reduce the informational asymmetry between the coalition partners and the minister party. Without informational advantages, it is more difficult for ministers to mislead their coalition partners, as the partners can acquire policy-specific information needed to evaluate the truthfulness of ministers’ statements.

### Oversight mechanisms in parliamentary democracies

Scholars highlight various control and oversight mechanisms in cabinet and parliament, which coalition partners use to lower the informational advantage of ministers and mitigate ministerial policy drift. At the cabinet level among others, these include coalition contracts (see e.g. Müller & Strøm, 2008; Bäck et al., 2017), ‘watchdog junior ministers’ (Lipsmeyer & Pierce, 2011; Thies, 2001), overlapping policy jurisdictions (Fernandes et al., 2016), cabinet committees and ‘inner cabinets’ (Andeweg & Timmermans, 2008; Müller & Strøm, 2000).

While cabinet-level oversight institutions play an important role for managing ministerial discretion, they have several limitations (see Martin & Vanberg, 2005, 2020). Cabinet posts are typically distributed proportional to party size and therefore smaller cabinet parties will not necessarily have enough ministerial capacity to monitor all (important) ministries held by their coalition partners (see Thies, 2001: 589). Further, coalition partners allocate cabinet posts with particular policy responsibilities, and oversight over other ministries has to be accomplished along with meeting their own ministries’ policy tasks. Given time constrains and their day-to-day workload, ministers and junior ministers might not be able to perform comprehensive oversight on most policy proposals and would need to prioritize.

In contrast, as Martin and Vanberg (2020) point out, the parliamentary stage gives coalition partners the opportunity to scrutinize *each* ministerial proposal, as every major policy initiative requires parliamentary approval. Every ministerial proposal undergoes several stages of scrutiny (committee sessions and parliamentary debates) and can, at least formally, be modified to contain ministerial drift and to enforce coalition compromise. Ministerial oversight in parliament might also be more efficient to coalition partners than cabinet oversight as they can revise ministerial bills and ensure the passage of the modified version at the same time which is not the case for cabinet-level decisions. In addition, parliamentary oversight as opposed to cabinet oversight can yield important political advantages for the coalition partners (Martin & Vanberg, 2020). Martin and Vanberg (2020) suggest that if ministerial proposals are modified, minister parties have political reasons to prefer that policy modifications happen after the bill enters the parliamentary stage, and not at the cabinet level. Usually, cabinet-level processes unfold behind closed doors and policy proposals get media attention after they are concluded and bills are ready for introduction to parliament. If intra-coalition control occurs only at the parliamentary stage, coalition partners can propose their desired policies, and use the media coverage to signal their commitment to their true policy position prior to any modifications (Martin & Vanberg, 2020).^1^ These advantages suggest that the parliamentary stage may be central for enforcing coalition compromise.

Parliamentary committees are central institutions which allow parties to reduce ministerial informational advantage (André et al., 2016; Carroll & Cox, 2012; Harfst & Schnapp, 2003; Kim & Loewenberg, 2005; Martin, 2004; Martin & Vanberg, 2004, 2005, 2011, 2020; Mattson & Strøm, 1995; Zubek, 2015). Scholars provide abundant empirical evidence that parliamentary committees are used for intra-cabinet oversight. For example, parliamentary committee chairs are appointed in a pattern which allows coalition parties to monitor their coalition partner in charge of the responsible ministry (Carroll & Cox, 2012; Kim & Loewenberg, 2005). Furthermore, the legislative process, of which committee sessions take a substantive part, lasts longer and results in more amendments when there is greater policy conflict between coalition partners (Martin and Vanberg, 2004, 2005). In a cross-country analysis of legislative organization, André et al. (2016) demonstrate that political systems where the risk of ministerial drift is high, have developed strong committees, which allow for policy specialization, information acquisition and oversight, as well as provide the possibility to amend proposals.

Parliamentary committees play a central role in the intra-cabinet oversight as they allow coalition partners to acquire policy-specific information, and to oversee and modify ministerial policy proposals. Committees typically have many ways to gather policy information and to monitor and check the policy actions of the corresponding ministries (Carroll & Cox, 2012; Kim & Loewenberg, 2005). They can schedule hearings, call witnesses, arrange open hearings, invite experts and interest groups, and request relevant documents from the executive (Martin & Vanberg, 2011). Committees which specialize in a particular policy area have cultivated relations with policy experts and have gathered extensive policy expertise over the years. This reduces the informational advantage of ministers and enables committees to propose feasible alternatives. Depending on the legislative rules, committees can sponsor amendments or directly rewrite ministerial proposals so that they reflect the coalition compromise position. Further, committees can use their policy expertise and amendment powers to bypass ministerial gatekeeping decisions and structure government policy output according to the overall coalition preferences. They can use the acquired policy information to correct ministerial bill content. Furthermore, committees with amendment powers can bring in additional reform measures in the form of amendments to ministerial bills and thus reduce ministerial gatekeeping powers.

Coalition partners (along with opposition parties) can also develop and introduce bills to the parliament on their own right. This option, however, has an important limitation. Without the administrative machinery of the responsible ministerial department, which has resources (civil servants, cultivated connections with interest groups and policy experts), and technical expertise, it is difficult for coalition partners not in charge of the responsible ministry to develop extensive bill drafts from scratch. Therefore, in practice, parties not in charge of a responsible ministry might be restricted to proposing modifications of already existing laws or bills.

Our discussion above suggests that, albeit with certain limitations, parliamentary committees provide the coalition partners with several opportunities to spot deviations from the coalition compromise, identify feasible alternatives to ministerial proposals and modify these proposals accordingly. They may even propose policies that ministerial gatekeeping has prevented from inclusion in government bills. Whether these opportunities are effective is an empirical question. If effective, these opportunities should increase the ability of coalition parties to mitigate ministerial drift in the proposed policies and restrict ministers’ gatekeeping powers.

### Oversight Mechanisms and Government Policy-Making

Given the central role of committee systems for intra-coalition oversight and government policy-making, the influence of ministers and the coalition as a whole should be contingent upon the committees’ ‘policing’ powers, which differ across countries (Harfst & Schnapp, 2003; Mattson & Strøm, 1995; Martin & Vanberg, 2011).

Ministerial policy expertise and the varying policing powers across political systems suggest that coalition parties which have greater ability to scrutinize their partners should have greater impact in the policy-making process. Therefore, the position of the coalition compromise should drive government policy output in parliaments with stronger policing powers. Conversely, ministers should be able to influence the policy-making process and thus, structure government policy output in parliaments with weaker policing powers. While scholars have paid much attention to whether and when coalition partners use various control mechanisms have analyzed the appointment pattern of oversight posts (Carroll & Cox, 2012; Fernandes et al., 2016; Kim & Loewenberg, 2005; Lipsmeyer & Pierce, 2011; Thies, 2001) and the duration and extent of parliamentary policing (Martin and Vanberg, 2004, 2005), so far, we know little about how effective oversight mechanisms are in their ability to reduce the informational advantage of ministers and constrain ministers’ policy influence.

Only recently have scholars shifted their focus to this question (see e.g. Fortunato et al., 2019). For example, Martin and Vanberg (2020) investigate how parliamentary policing strength conditions the impact of coalition compromise and ministerial preferences on policy changes in the labour policy area, more specifically, changes in unemployment benefits across countries and time. They provide direct evidence that policing institutions are substantive for the direction of policy changes, showing that in parliaments with strong policing powers, policy changes are driven by the compromise position in cabinet. In parliaments with weak policing powers, policy changes reflect the preferences of the minister party in charge. Hence, parliamentary oversight mechanisms seem to be effective in their ability to correct ministerial drift and bring proposed policies closer to the coalition compromise.

Martin and Vanberg’s (2020) work breaks new ground and sets the first steps in the study of how legislative oversight institutions influence government policy choices. While their approach allows the evaluation of the impact of legislative oversight institutions on ministers’ ability to shape the *substance* or *direction* of government policy choices, it remains unclear whether parliamentary oversight mechanisms also constrain ministerial ability to decide what to initiate (positive agenda power) and what to keep off the government agenda (gatekeeping powers). We aim to narrow this gap in the literature.

## Theoretical Framework

### Expectations About Governmental Reform Making

In order to analyze the impact of ministerial autonomy and the coalition as a whole on government policy-making we propose to focus on the number of important reform measures passed by the government. We start from the simple and well-established theoretical expectation that if the current government is dissatisfied with the policy status quo, then this government will try to introduce policies to change the policy status quo and move it closer to their desired position. Therefore, the further away the location of the policy status quo from the policy preferences of the actor(s) who shape government policy choices, the more policy changes should the actor(s) pass to bring the policy status quo closer to the preferred policy position. Accordingly, the number of policies a government produces, which change a policy status quo, should be systematically related to the discrepancy between the position of the status quo policy and the policy position desired by the actors who drive government decisions.

We make two central assumptions. First, we assume that parties are driven by policy-seeking goals.^2^ Second, governments inherit a policy status quo, which to a great extent is shaped by the actions of preceding government(s). We therefore represent the position of the status quo through the position of the previous government(s). Accordingly, we expect that a large discrepancy between the position of the current and previous government(s) in a given policy area should result in a high number of reform measures. Small discrepancies should results in a low number of reform measures aimed at changing the policy status quo.

**Figure 3.**
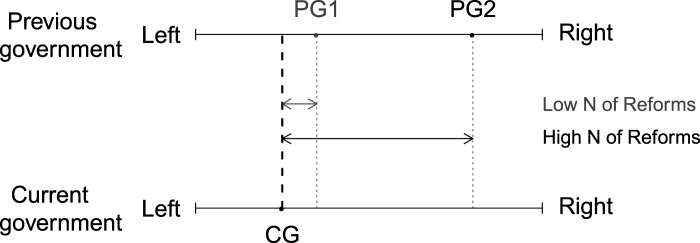
Government alternation and number of important reforms.

We illustrate this logic in Figure 3. It depicts the position of the current government (CG) and the representation of the status quo through the location of the previous government on the left–right scale (PG). We show two scenarios. In the first scenario, the distance between the current government (CG) and the previous government (PG1) is small. Accordingly, here we expect a low number of reforms that change the inherited policy status quo. The second scenario depicts a situation, where the location of the previous government (PG2) and thus the inherited policy status quo is further away from the position of the current government (CG). Therefore, here we would expect a high number of policy reforms that change the policy status quo.

We do not know whether ministers, or the coalition as a whole prevail in government policy-making and drive the final government policy output and thus government policy position. However, the strength of the systematic relationship between the alternation of policy preferences of a given actor (responsible minister or the coalition as a whole) and the number of reform measures that change the policy status quo should reveal who has a stronger influence over government policy choices. In particular, if the preferences of the minister party in charge drive government policy, then the discrepancy between the policy preferences of the current and previous responsible minister parties in a given policy area should be systematically related to the number of policy changes the current government produces. Greater difference should result in more policy changes, whereas smaller difference should result in fewer policy changes.

For example, suppose that in a world where ministers determine output in the policy area of their purview, Conservatives controlled the Social Affairs Ministry in the previous government. Also, suppose that the Social Democrats control the Social Affairs Ministry in an incoming government. In such a scenario, it is highly likely that the Social Democrats will want to change the policy status quo they inherited, and are thus likely to propose a number of reforms that will move the social policy position away from the status quo and closer towards their preferred policy position. In this scenario, if the preferences of the minister party drive government policy choices, an alternation in the responsible minister party would imply a left-wing shift in the department’s policy, which should result in the adoption of a number of policies.

If instead, the position of the coalition compromise drives government policy choices, the discrepancy between the policy position of the current and previous coalition compromise should be systematically related to the number of policy changes the current government produces. In particular, greater difference should result in more policy changes, smaller difference should result in less policy changes. We capture the changes in the preferences of the responsible minister party by taking the distance between the positions of the responsible minister party in the current and preceding cabinets and label this *responsible minister alternation*. Similarly, we capture the changes in the policy position of the coalition as a whole by taking the absolute distance between the seat-weighted and saliency-weighted compromise in the current and preceding governments and label this *coalition alternation*. Given our above discussion, we propose the following two hypotheses:

**Hypothesis 1** (Responsible Minister Alternation): *Governments pass more reform measures the greater the policy distance between the ideal positions of the responsible minister party in the current and previous cabinet(s)*
**Hypothesis 2** (Coalition Alternation): *Governments pass more reform measures the greater the policy distance between the ideal positions of the seat-weighted and saliency-weighted compromise of the current and the previous cabinet(s).*

### Expectations About Institutions and the Agenda-Setting and Gatekeeping Powers of Ministers

Our discussion of the policy-making process in coalition governments suggests that the extent to which the preferences of the responsible ministers, or the coalition as a whole, structure government policy decisions, depends upon the institutional setting within which coalitions govern. This should, in particular, be dependent on institutional features that determine if coalition partners can use effective oversight mechanisms to reduce ministerial informational advantages and adopt amendments that mitigate ministerial policy drift. Given that parliamentary oversight mechanisms have several advantages over cabinet oversight mechanisms, in the ensuing part of the paper we focus on the parliamentary arena, in particular, the oversight strength of legislative committee systems. We suggest that this focus allows us to disclose to what extent the agenda-setting and gatekeeping powers of ministers are constrained by the coalition partners in different institutional settings.

What policy outputs governments ultimately produce depends upon several interrelated decisions: (1) what ministers decide to propose and what to shelve; (2) which policies coalition partners will manage to bring in through committee amendments as part of a given legislative proposal or draft on its own; and (3) which of the proposed policies will get the necessary parliamentary majority and thus, the support of the coalition parties in majority governments.

We expect that ministers will develop and propose policies from which they expect their party to benefit and refrain from introducing and agreeing to policies that they deem politically costly. Legislative policy oversight can influence the cost-benefit calculations of ministers in a substantive way. If coalition partners have limited opportunities to acquire policy-specific information, develop feasible alternatives and modify ministerial policy proposals, then responsible ministers can propose policies, which are close to their own policy position and include all desired and feasible policies from their agenda. Without policy expertise and current policy-specific information, coalition partners will have a hard time to dispute ministers’ claims that these proposals are the ‘best possible’ policy alternative to the policy status quo given the current policy situation. In such a scenario, the number of passed government policy issues *should be systematically related* to the alternation in ministerial policy preferences.

In contrast, in parliaments with strong policing powers, coalition partners can evaluate ministers’ feasibility arguments and decide to reject or substantively modify ministerial proposals so that they are closer to the coalition compromise position. Both scenarios are highly costly for the responsible minister and therefore ministers might try to avoid them whenever possible. Policy rejection carries high opportunity and audience costs. Substantive bill amendments might shift the policy further away from the responsible minister party. Such shifts carry electoral costs, because in such instances minister parties have effectively yielded to substantive policy concessions and hence compromised their electoral policy promises.^3^

Studies show that while voters might have a preference for cooperation between parties, they also desire that their party’s preferences prevail (Harbridge & Malhotra, 2011). Fortunato (2019) finds that voters punish incumbent parties for higher levels of coalition compromise. In the face of such costs, minister parties are better off shelving highly contested policy matters. We expect that in parliaments with strong policing powers, ministers anticipate substantive amendments or policy rejection and therefore refrain from introducing some policies from their policy agenda. Accordingly, in such instances the number of government policies should be less systematically related to the alternation of ministerial policy preferences. Another reason why the number of government policies in parliaments with strong policing powers might be less systematically related to the alternation of ministerial preferences, is that coalition partners are able to bring in policies which correspond to the policy agenda of the coalition as a whole.

Accordingly, we identify two ways in which parliamentary oversight helps coalition partners constrain ministerial agenda-setting power; indirectly – ministers anticipate scrutiny and the associated costs and refrain from introducing desired but highly contested policies; and directly – coalition partners use committee’s policy expertise to identify feasible policy issues which were omitted in the ministerial proposal and bring these in through bill amendments, by demanding from the responsible minister to propose these or by drafting new proposals on their own. Both ways should lead to a *weaker* systematic relationship between the number of government policies and the alternation of ministerial preferences. Thus, we expect that when coalitions operate in parliaments with strong (weak) legislative oversight mechanisms, in particular, where coalition partners can gather policy-specific information, scrutinize and amend ministerial proposals, responsible ministers will have a weaker (stronger) impact on the policy output of governments. We use the time-variant legislative policing strength index developed by Martin and Vanberg (2011, 2020) for this purpose and hypothesize that:

**Hypothesis 3** (RM Alternation and Institutional Context): *The impact of responsible minister alternation on the number of reform measures decreases with greater legislative policing strength.*

Hence, there are two reasons for the limited impact of ministers on government policy output in parliaments with strong legislative policing powers: first, ministers themselves may decide to keep desired but highly disputed policies off the government agenda, and second, coalition partners may gather sufficient policy information to modify ministerial proposals and bring in desired and omitted policy issues. The differentiation between the two scenarios is of substantive importance. In the former case, ministers refrain from introducing policies that are not agreed with their coalition partner(s), and therefore face certain limitations what they ultimately propose from their desired policy agenda. In the latter case, coalition partners actively structure the government’s policy agenda by bringing in policy issues suppressed by the responsible minister themselves. As ministers can shelve policies representing the coalition compromise, because they are party-politically costly, the ability of coalition partners to bring in such policies themselves should invalidate these gatekeeping decisions of ministers.

We can disentangle the two mechanisms by also analyzing whether parliamentary policing institutions help the coalition as a whole to structure policy output. If legislative oversight helps coalition partners to bypass ministerial gatekeeping decisions by allowing them to bring in desired policies themselves, we should find that the impact of the coalition compromise preferences increases with greater legislative policing powers. If policing institutions in parliament allow the coalition as a whole to constrain ministerial agenda-setting *and* gatekeeping powers, and thus manages to structure governmental policy output, then we should find that the impact of coalition alternation on the number of important government reforms increases with greater legislative policing strength. Thus, we hypothesize that:

**Hypothesis 4** (Coalition Compromise and Institutional Context): *The impact of coalition alternation on the number of reform measures increases with greater legislative policing strength.*

## Data and Empirical Design

### A Comparative Data Set on Important Reform Measures

To investigate the role of legislative oversight mechanisms for the impact of minister and coalition preferences on government policy output, we use a newly constructed dataset with *important* policy *reform measures* passed in the social and the economic policy domains in nine Western European countries over a period of 20 years (1985–2005).^4^

Our theoretical expectation suggests that when parties face a policy status quo far away from their ideal point, they have incentives to change it. Previous studies measure a change to the policy status quo either by looking at the change in the substance or direction of a given policy issue, for example, changes in unemployment benefits (Martin & Vanberg, 2020), or by analyzing the number of *significant* laws enacted (Tsebelis, 1999, 2002), which by definition change the policy status quo. The former approach provides a more precise measure of policy change but necessitates a fine-grained evaluation of the policy issue at different points of time, which is difficult to achieve for many policy issues. The approach of analyzing significant laws allows for a broader coverage of different policy areas but comes with the challenge of identifying which laws are significant. It has been achieved either for specific policy issues (e.g. labour laws; see Tsebelis, 1999), or for specific countries (e.g. Conley & Bekafigo, 2010). However, laws can regulate one policy issue (e.g. minimal wage) or provide a package of issues in a given policy area (e.g. combine unemployment benefits with working time regulations). The variation of the number of policy issues in a given law across time and across countries with different legal cultures makes a direct comparison of significant *laws* problematic.

To overcome these challenges – broad coverage of policy issues and comparability of cases – we propose to concentrate on *significant reform measures* passed by the government. A reform measure is a significant change of one policy parameter, for instance, an increase of the retirement age, a cut in unemployment benefits for long-term job-seekers, a relaxation of restriction on retail opening hours, a ban on insider trading or the introduction of an investor bonus. Moreover, given that committees can include *individual* policy issues or policy measures via amendments of bill proposals, it is the more critical to investigate *individual* issues and not entire laws as a unit of analysis.

To gather our reform data, we rely on regular country reports issued on a quarterly basis by the *Economist Intelligence Unit* (EIU). Following a detailed coding scheme, we coded more than 1000 periodical country reports issued by the EIU, from which we extracted more than 2000 important reform measures adopted in nine Western European countries with coalition government experience, over a period of 20 years (ca. 1985–2005, covering the entire office periods of the cabinet beginning or ending closest to these dates). Our dataset includes reform measures from two broad policy domains – economic and social policy, covering reform measures from 11 policy categories (Product Market and Services, Capital Market, Monetary Policy, Budget/Public Debt, Old-Age/Disabled/War Victims, Sickness and Maternity, Long-term Care, Unemployment, Family, Social Welfare and Social Security), and around 110 sub-categories^5^ for the following countries: Austria, Belgium, Denmark, Finland, France, Germany, Ireland, Italy and the Netherlands.

The periodical country reports by the EIU provide a unique opportunity to extract important reform measures issued by the government over time. The EIU is a business unit of the Economist Group with more than 70 years of experience in policy research, analysis and forecasting for countries around the globe. The EIU regularly issues country reports (prepared by full-time employed country specialists), which provide comprehensive information about the current policy status quo and concrete policy changes in a given country. EIU’s customers range from business investors, international organizations, government agencies, to academic institutes. EIU’s goal and target group ensure that the country reports include only important reforms that change the status quo and exclude incremental provisions. Further, the EIU reports concrete policy changes and provides detailed information about *individual* important reform measures included in a given law, instead of just mentioning the law.^6^

We code individual reform measures, even if they occur in packages in a given law, and we code only those reform measures that indicate a policy change and specify the policy instrument used to introduce this policy change. For example, vague phrases such as ‘the government has started to fight rising levels of unemployment’ are *not* coded because they do not indicate a specific action taken or a specific instrument introduced to reduce unemployment. In contrast, we code statements such as ‘For this reason the budget proposal (…) lowered the level of [unemployment] benefit from 90% to 80% of total pay’ (EIU, 1993: 15) or “New legislation allowing for a gradual relaxation of restrictions on retail opening hours, in particular on Sundays, came into force in July 2005” (OECD, 2006: 55) as one measure.^7^

### Variation in reform productivity across the Western European countries

**Figure 4.**
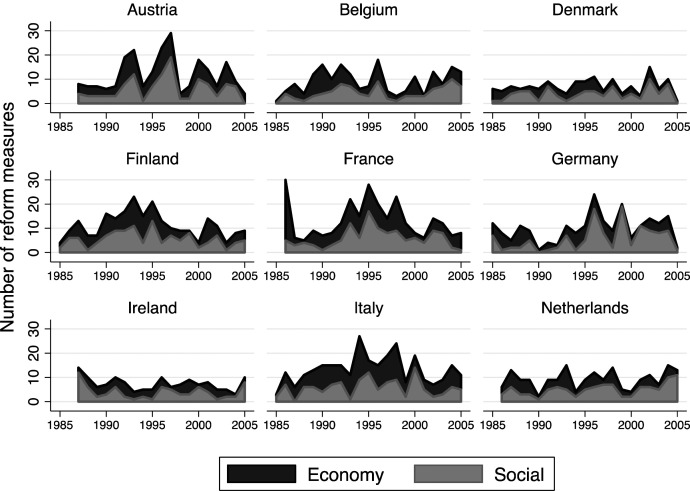
Number of social and economic government reform measures across countries and time.

Since we aim to analyze the role of minister parties and the coalition as a whole for governmental policy output, our outcome variable (dependent variable) is the *number of important reform measures* adopted in a given coalition cabinet and policy area. Figure 4 presents the distribution of the number of reform measures by policy area – social and economic policy over time for each country in the sample.

There is considerable variation in the number of reform measures within and between countries. As a ‘test’ of face validity, we can find distribution patterns that correspond to central trends and critical events in given countries. Pronounced temporary slumps in reform activity, such as, for example, in Austria in 1994 and 1995, in Belgium in 1995 and 1999, in Germany in 1990 and 1998, in France in 1988, 1997 and 2002, and in Italy in 1994 and 2001, are most likely due to general elections, when governments have less time to pass reforms due to parliamentary dissolution and the government formation process.

Related to our research question, we can see that the alternation of governments, and more specifically responsible ministers, seems to be followed by higher reform productivity. For example, in France, the conservative party UDF took over the social affairs ministry from the Socialist Party after the elections in 1993 and we can see a considerable peak in the number of social policy reforms after 1993. We find a similar pattern in Germany. A long-lasting conservative-liberal government between CDU/CSU and FDP was replaced by a leftist government between the SPD and the Greens in 1998. Here the responsibility for social policy moves from the centre-right CDU/CSU to the centre-left SPD, which is followed by a considerable peak in the number of passed social policy reform measures.

### Measuring Responsible Minister and Coalition Compromise Alternation

Our main explanatory variables are *responsible minister alternation* and *coalition alternation*, which implies that we need information on the policy positions of responsible ministers and the coalition compromise. There are several ways to measure parties’ or politicians’ policy preferences, including mass surveys, expert surveys, elite surveys, political text, roll-call votes and speeches (see e.g. Benoit & Laver, 2006). The most widely used data source that includes information on party policy preferences and varies across countries and time is the MARPOR dataset, based on a hand-coding of election manifestos (Volkens et al., 2015).

The manifestos data is the most appropriate source for our purposes. Party manifestos reflect the electoral commitments of parties and their desires to maintain or change the inherited policy status quo. Since party manifestos are updated from one election to another, the MARPOR data is also well suited to capture the variation of party positions over time and thus fits well with our time-variant data structure. We focus on the social and economic policy areas and calculate the positions of each party responsible for these policy areas on the socio-economic left–right dimension based on 14 categories applying the logarithmic scaling introduced by Lowe et al. (2011) (see Appendix D (Supplementary Material) for details on the categories and the formula we used).

For simplicity, we assume that parties are unitary actors and approximate the position of the responsible minister with the ideal position of the minister’s party. We identify the responsible minister party for the social and economic policy areas using the data provided by Seki and Williams (2014). Their data set draws on the *Political Data Yearbook* of the *European Journal of Political Research* and covers the 1991–2014 period. We extend these data for the years prior to 1991 using an online database,^8^ which we crosscheck with available information from the official webpages of national governments.

There are some occasions of ministerial reshuffles. Often these reshuffles are changes of a party’s personnel while the same party remains in charge of the ministry. In some cases, however, the responsibility for a given ministerial department is moved from one government party to another. To account for these changes, we calculate the *time-weighted average position* of the responsible minister party(ies). In particular, the positions of all parties in charge of a given ministerial department are weighted by the share of time they controlled the portfolio.

Our theoretical argument suggests that if responsible ministers and their parties can influence governmental policy output in the policy area in their jurisdiction, then the inherited *policy* status quo should to a large extent reflect the position of the responsible minister party from past cabinet(s) and should hence motivate incoming ministers with a different party background to greater activism to change that status quo. To capture this, we take the time-weighted average position of the minister parties that were in charge of a given policy area for a period of 4 years prior to the current government. This measure allows the position of minister parties with shorter time spans to contribute less to the location of the overall policy status quo. We choose 4 years to cover the standard duration of one full legislative term.

We calculate the *responsible minister alternation* variable for each policy area separately by taking the absolute distance between the current minister’s position (the time-weighted average position of all minister parties in charge of the policy area in the current government) and the time-weighted average position of all minister parties in charge of a given policy area for a period of 4 years prior to the current government.

Further, our theoretical argument suggests that if the coalition as a whole influences governmental policy output in a given policy area, then the inherited *policy* status quo should to a large extent reflect the coalition compromise position from past cabinet(s) and should hence motivate incoming governments with a different coalition compromise policy position to greater activism to change the inherited policy status quo.

We calculate the coalition compromise position in the current government as the *seat-weighted and saliency-weighted average position* of all parties in the current government.^9^ In addition, we calculate the seat-weighted and saliency-weighted compromise position of all preceding governments for a period of 4 years prior to the current government and take the time-weighted average of these. We measure the *coalition alternation* variable by taking the absolute distance between seat-weighted and saliency-weighted compromise in the current government and the time-weighted average of the seat-weighted and saliency-weighted compromise positions of all cabinets for a period of 4 years prior to the current government. To construct this measure, we use data provided by ParlGov (Döring & Manow, 2016), which includes information on cabinet composition and seat distribution of government parties. We calculate the saliency weights for the socio-economic left–right dimension for each party based on 14 categories applying the logarithmic scaling for saliency (Lowe et al., 2011) (see Appendix D (Supplementary Material) for details on the policy categories and the formula we used).^10^

### Measuring the Strength of Legislative Institutions

**Figure 5.**
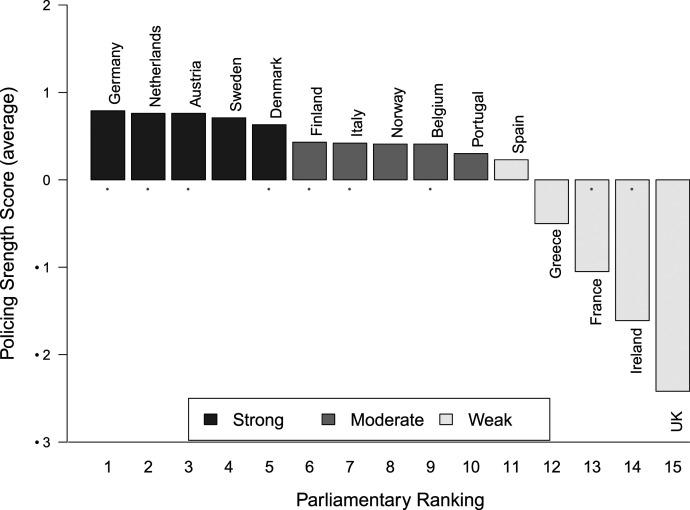
Legislative policing strength index – country average values. Note: Figure 5 depicts the average values of the legislative policing strength index per country (Martin & Vanberg, 2020). Political systems with strong (weak) legislative institutions have high (low) policing index values. Dots mark the countries included in our analyses. *Source.* Martin and Vanberg (2020).

Martin and Vanberg (2011, 2020) highlight the importance of eight features that strengthen or weaken coalition partners in their ability to subject responsible ministers to oversight, develop viable alternatives and amend ministerial proposals. These include the number and size of legislative committees; whether committees correspond to cabinet ministries; whether there is a binding plenary debate before committee stage; if committees have the right to compel witnesses and documents and rewrite draft bills directly; and whether ministers can unilaterally reject amendments, force take-it-or-leave it options on ministerial draft bills and declare a bill ‘urgent’, which restricts legislative debate and makes parliamentary oversight less effective. Martin and Vanberg (2020) create a time-variant policing strength index that varies by country and year. For our analysis, we create a time-weighted average for each cabinet. We use this legislative policing strength index in our analysis and interact it with our responsible minister alternation and coalition alternation variables. We show the average values for each country in Figure 5 and depict the time-variant values by cabinet in Figure E2 in the Appendix (Supplementary Material).^11^

In political systems with ‘strong’ legislative institutions, indicated by a higher policing strength index (e.g. Germany, Netherlands, Austria and Denmark), coalition partners should be able to influence policy-making. Where legislative institutions are ‘weak’, indicated by a lower value on the policing strength index (e.g. Ireland and France), coalition partners should be unable to influence policy, and thus the preferences of the responsible minister party should be decisive (Martin & Vanberg, 2020).

### Operationalization of Control Variables

We also include several control variables, for example, controlling for the number of cabinet parties, as well as for coalition conflict. We measure potential coalition conflict by taking the absolute distance between the seat- and saliency-weighted coalition compromise position and the responsible minister party in the current government. Time is an important resource for governments who aim to introduce reforms. Given that cabinets can have different life spans, our models also control for cabinet duration (measured in years).

To identify the effects of our explanatory variables, we control for three macro-economic factors that we view as potential confounders: the level of unemployment, gross domestic product (GDP) growth and inflation. We expect that these factors might influence both, the number of reform measures a government produces and the changes in the policy positions of government parties. For example, high levels of unemployment might mark a crisis, which may trigger the replacement of the incumbent government by a new government, which will respond by reforming existing and introducing new social welfare provisions. Similarly, economic deterioration can necessitate government change as well as result in more economic reforms. These macro-economic factors capture the demand and ability for policy change.

Our data follows a cabinet-level structure and therefore we aggregate the socio-economic data to the cabinet level. In particular, we calculate the *average* percentage change in GDP growth and inflation from one year to another in a given cabinet. To capture a crisis due to unemployment, we first calculate the average unemployment level for a country during a given cabinet and then create a dummy taking the value 1 when the average cabinet unemployment level is high (larger than 11.7% – mean plus one standard deviation) and 0 otherwise. Here we use OECD data compiled in the Comparative Political Dataset by Armingeon et al. (2015). Table 1 presents descriptive statistics for all variables included in our models.

**Table 1.** Descriptive Statistics.

	Mean	Standard deviation	Minimum	25th %	Median	75th %	Maximum
Number of reform measures (DV)	12.68	8.68	0.00	6.00	11.50	17.00	37.00
Responsible minister alternation (RMA)	0.97	0.83	0.00	0.31	0.79	1.38	3.92
Coalition alternation (CA)	0.78	0.74	0.02	0.25	0.63	1.04	3.92
Legislative policing index	0.27	0.74	−1.91	0.35	0.46	0.66	1.01
Number of cabinet parties	3.35	1.44	2.00	2.00	3.00	4.00	9.00
Responsible minister – coalition distance	0.52	0.47	0.00	0.14	0.38	0.83	2.17
Average Δ in GDP growth	2.43	1.80	−2.75	1.55	2.39	3.28	9.90
Unemployment crisis (0/1)	0.08	0.27	0.00	0.00	0.00	0.00	1.00
Average Δ in inflation rate	2.75	1.57	0.30	1.76	2.28	3.40	8.96
Cabinet duration (years)	2.51	1.41	0.11	1.12	2.56	3.86	5.02
Observations	150						

## Empirical Analysis

Given that our dependent variable – the number of reform measures adopted by a given cabinet in a given policy area – is a count variable which is overdispersed, we use negative binomial regression models (Long, 1997). A common concern for most cross-sectional data is country-level heterogeneity. Two widely used ways to account for this is to estimate multilevel models with country random effects (see Gellman & Hill, 2007) or estimate models with country dummies (fixed-effects models). We run multilevel mixed-effects negative binomial models with random intercept by country and fixed effects by policy area in our main analyses. Additionally, as a robustness check, we run models with fixed effects by country and policy area and clustered standard errors by country (see results in Table E6 in the Appendix (Supplementary Material)). Both, the multilevel and the fixed-effects models yield substantively similar results.

### Analyses of Ministerial and Coalition Compromise Alternation and Reform Productivity

Table 2 presents the results from three multilevel mixed-effects negative binomial models. The estimated coefficients are exponentiated to reflect incidence rate ratios, where values higher (lower) than 1 stand for a positive (negative) effect of the covariate on the dependent variable.

**Table 2.** Multilevel Mixed-Effects Negative Binomial Models of the Number of Social and Economic Government Reforms in Coalition Governments.

	Model 1	Model 2	Model 3
RMA	1.12^†^	1.17^∗^	1.04
	(0.07)	(0.08)	(0.07)
CA	0.93	0.86^∗^	0.95
	(0.07)	(0.06)	(0.07)
Legislative policing index		1.27^∗^	1.10
		(0.15)	(0.10)
RMA^∗ ^legislative policing index		0.78^∗∗∗^	
		(0.05)	
CA^∗^ legislative policing index			0.79^∗∗∗^
			(0.06)
Number of coalition parties	1.00	1.03	1.03
	(0.04)	(0.03)	(0.03)
Responsible minister-coalition distance	1.18^†^	1.13	1.22^∗^
	(0.10)	(0.09)	(0.10)
Average Δ in GDP growth	0.93^∗∗^	0.92^∗∗∗^	0.92^∗∗^
	(0.02)	(0.02)	(0.02)
Unemployment crisis (dummy)	1.14	1.18	1.11
	(0.19)	(0.20)	(0.19)
Average Δ in inflation rate	0.95^†^	0.96	0.95^†^
	(0.03)	(0.03)	(0.03)
Cabinet duration (years)	1.61^∗∗∗^	1.61^∗∗∗^	1.61^∗∗∗^
	(0.05)	(0.05)	(0.05)
Social (vs. economic) policy	1.00	0.99	1.01
	(0.07)	(0.07)	(0.07)
ln(alpha)	0.11^∗∗∗^	0.09^∗∗∗^	0.10^∗∗∗^
	(0.02)	(0.02)	(0.02)
Random intercept (country level)	1.03	1.01	1.01
	(0.02)	(0.01)	(0.01)
Observations	150	150	150
Log likelihood	−450	−443	−444
AIC	923	914	916

RMA: responsible minister alternation; CA: coalition alternation; AIC: Akaike information criterion.

Note: Multilevel models with varying intercept by country and fixed effects by policy area; exponentiated coefficients with standard errors in parentheses (^†^*p* < 0.10, ^∗^*p* < 0.05, ^∗∗^*p* < 0.01, ^∗∗∗^*p* < 0.001); dependent variable: number of government reform measures per country-cabinet and policy area.

We first run a baseline model, where we test the effect of responsible minister alternation (Hypothesis 1) and coalition alternation (Hypothesis 2) on the number of important reform measures with varying intercept by country and fixed effects by policy area. In addition to both alternation variables, Model 1 includes coalition conflict, number of cabinet parties, cabinet duration and social-economic factors. Model 1 shows that the responsible minister alternation variable is positive and statistically significant at the 0.1 level. If we increase responsible minister alternation by one unit, the number of passed reform measures increases by 12 percentage points. In contrast, the effect of coalition alternation on the number of government reform measures is not statistically significant and is negative, contrary to our expectations.

Model 1 tests the overall impact of responsible minister alternation and coalition alternation on the number of government reform measures without taking into account the institutional context. However, the impact of minister parties and their coalition partners on government policy output should depend upon the institutional context, in particular, upon the parliament’s policing strength. To test Hypothesis 3, where we suggest that parliamentary oversight should constrain minister’s impact on the government policy output, we interact our responsible minister alternation variable with the policing index developed by Martin and Vanberg (2011, 2020). We present our results in Model 2 and find strong support for Hypothesis 3. The interaction effect between responsible minister alternation and the policing index is, as expected, negative and statistically significant. The effect of responsible minister alternation is positive and substantively high for low policing strength and decreases to zero with higher values of policing strength. These results support the suggestion that legislative oversight constrains ministerial impact on government policy output, giving clear support for Hypothesis 3.

**Figure 6.**
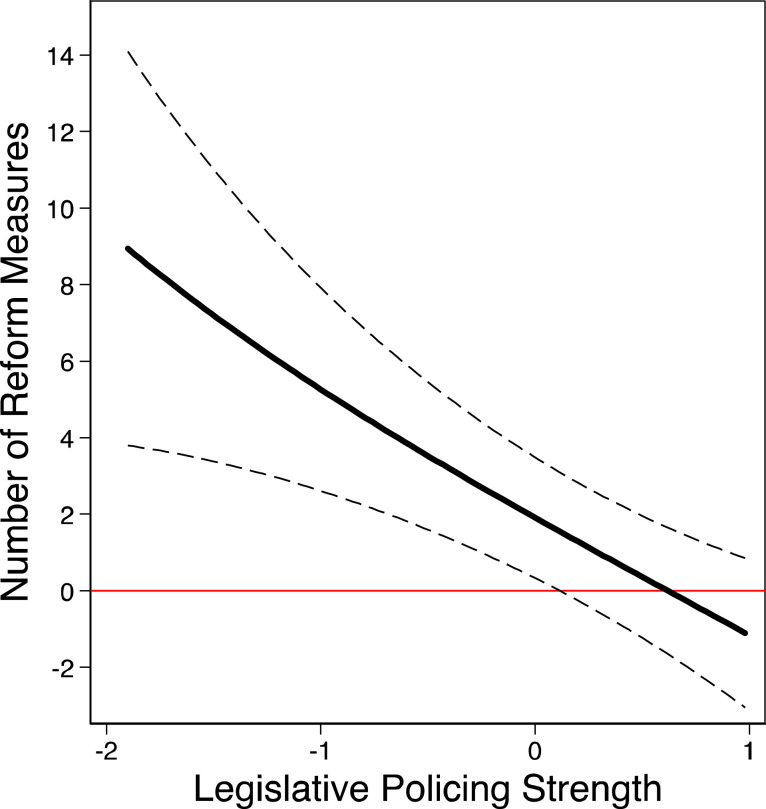
Average marginal effect of responsible minister alternation on the number of social and economic government reform measures for different levels of legislative policing strength. Note: Expected change in the number of important government reform measures given one-unit change of responsible minister alternation for different levels of legislative policing strength with 95% confidence bounds (based on Model 2, Table 2).

We present our substantive findings in Figure 6, which shows the effect of one-unit increase in responsible minister alternation on the number of important reform measures for different levels of policing strength from Model 2. Here we can see that a one-unit increase in the responsible minister alternation in legislatures with weak policing strength (e.g. Ireland with policing strength equal to −1.9 in the 1980s and early 1990s) results in about nine more reform measures in a given cabinet and policy area. Given an average of around 12.7 reform measures, an increase of nine reform measures constitutes about 70% higher government reform output as a result of one-unit increase in responsible minister alternation. In contrast, in ‘strong’ parliaments (e.g. Austria (1.0 for 2000–2005)), Germany (0.97 for 1999–2002) and Netherlands (0.95 for 1990–1993), we find that a one-unit increase in the responsible minister alternation has practically no effect on government reform productivity. In particular, it results in a decrease of about one reform measure in the governmental reform output, whereby the 95% confidence intervals of these effects include 0.^12^

### Analyses of Legislative Policing Institutions and Reform Productivity

While our results reveal that ministers can structure government policy output according to their policy preferences in legislatures with weak policing institutions and are constrained to do so in parliaments with strong policing institutions, it is essential to analyze why this is the case – because the responsible minister party shelves policies or because the coalition as a whole drives government policy output. Earlier, we proposed that parliamentary oversight allows coalition partners to bypass ministerial gatekeeping decisions and bring in desired policies on their own. If coalition partners are able to utilize this opportunity and the coalition as a whole drives government policy decisions, we should observe that the effect of coalition alternation *increases* with higher legislative policing strength (Hypothesis 4).

**Figure 7.**
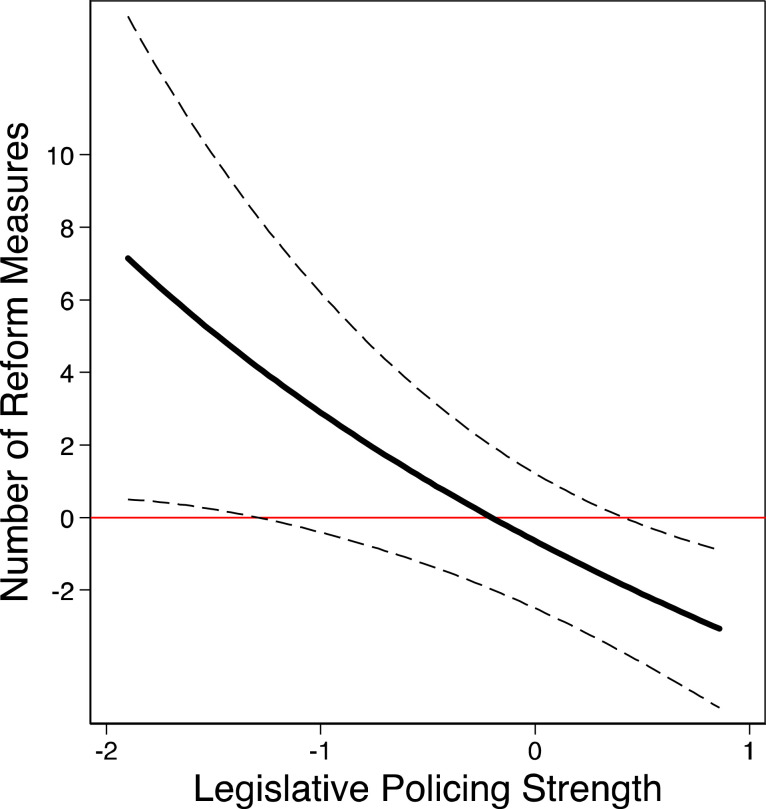
Substantive effect of coalition alternation on the number of social and economic government reforms given different levels of legislative policing strength. Note: Expected change in the number of government reform measures given one-unit change of coalition alternation for different levels of legislative policing strength with 95% confidence bounds (based on Model 3, Table 2).

We test this expectation in Model 3. The interaction effect between coalition alternation and the legislative policing index is negative (below 1) and statistically significant. This result runs contrary to the relationship expected in Hypothesis 4. We present the substantive effect size of coalition alternation for different policing strength in Figure 7. Here we can see that the average marginal effect of a one-unit increase in coalition alternation in legislatures with weak policing strength (−1.9) results in about seven more reform measures in a given cabinet and policy area. In contrast, in ‘strong’ legislatures, we find a *decrease* in the number of government reform measures and the effect of coalition alternation on the number of passed reform measures is even negative in very strong legislatures.

Our results from Models 2 and 3 reveal that while the effect of responsible minister alternation *decreases*, the effect of coalition alternation does *not increase* with greater legislative policing strength. These findings suggest that although policing institutions constrain minister parties in their agenda-setting decisions, the ability of coalition partners to correct ministerial decisions and actively structure policy output at the parliamentary stage is limited. More specifically, the decreasing effect of coalition alternation with stronger policing powers implies that the reason why ministers have weaker impact on the government’s policy agenda when policing is strong, is *not* that coalition partners actively structure the government agenda, but rather that minister parties strategically shelve policy proposals in anticipation of rigorous parliamentary scrutiny and amendments.

Strategic gatekeeping by responsible ministers can also explain the positive effect of coalition alternation in ‘weak’ legislatures and the negative effect of coalition alternation in ‘strong’ legislatures. In legislatures with *weak* policing opportunities, responsible minister parties might be willing to propose policy issues from the agenda of the coalition partners or the cabinet as a whole. In such institutional settings, ministerial proposals get less oversight, if any, and ministers can use their informational advantage to determine the form and substance of the draft bill and of the final law. Ministers might prefer to initiate a policy issue desired by their coalition partners today, as they can place it closer to their own policy preferences, rather than shelve it for the future, when it might land further away from the preferences of the minister party. In contrast, in settings with *strong* legislative policing powers, ministers have weaker incentives to draft and initiate policies from the agenda of the coalition partners or the cabinet as a whole. They can anticipate strict scrutiny and expect that proposals further away from the coalition compromise will either fail to pass or will be massively modified. Both scenarios are highly costly for the responsible minister. In the face of such costs, minister parties may be better off to shelve highly contested policy matters.

## Conclusions

The seminal work of Laver and Shepsle (1990, 1996) has shifted coalition studies from an exclusive focus on the beginning and end of the coalition life cycle towards paying attention to what comes in-between, making coalition governance a crucial determinant of both cabinet formation and duration. They also offered a parsimonious theory of coalition governance that characterized individual ministers as ‘policy dictators’ in their jurisdictions. Yet, the theory of ministerial government conflicts with the many attempts coalition parties make to design and employ various control and oversight mechanisms in parliament and cabinet. A growing literature on coalition governance institutions thus has taken issue with the ‘policy dictator’ view of coalition governance (e.g. Müller and Strøm, 2000; Strøm et al., 2008, 2010).

A literature has begun to emerge that seeks to evaluate the role of ministers in policy-making and the effectiveness of oversight mechanisms. One approach focuses on actions taken by the coalition parties in parliament. Studying legislative amendments, Martin and Vanberg (2014) show that parties indeed use ministerial discretion to shape the proposals of their portfolios in a partisan fashion while their coalition partners use parliamentary instruments to ‘correct’ them. Another approach focuses on policy content and changes in the party control of ministries. For example, Martin and Vanberg (2020) show that changes in the responsible minister party lead to important policy shifts in the direction of the party’s ideal position, if not prevented by some other mechanism in the coalition’s system of checks and balances.

Our approach builds on these works and contributes a new perspective and new comprehensive comparative data. Rather than investigating outcomes in terms of policy contents or activities of mutual coalition control, we focus on important government policy reform measures, that is, individual changes of important policy parameters such as specific social policy benefits or investor incentives, and the minister in charge. Our analysis shows that holding a ministry provides the respective party with the power to foster its partisan policy priorities. In line with the previous literature, this finding supports the intuition underlying the idea of ministerial government, namely, that coalition parties use opportunities to implement their partisan rather than the joint coalition agenda when they arise. We find that such agency losses become insignificant when strong legislative institutions allow coalition partners to monitor each other. Collectively, Martin and Vanberg’s (2020) conclusions, and our findings, suggest that coalition governance works if appropriate institutions are in place. If not, significant deviations in the direction favoured by the responsible minister’s party do occur.

Furthermore, and this has not been shown in previous work, our results suggest that although parliamentary oversight constrains ministerial policy choices, in particular, what to propose from their desired policy agenda, ministers are still effective as gatekeepers and can shelve policies desired by their coalition partners. In other words, our results suggest that while parliamentary oversight constrains the agenda-setting decisions of minister parties, it is less effective in bypassing ministerial gatekeeping decisions and provides limited opportunities for coalition partners to structure policy output according to their desired policy agenda.

This finding has important implications for democratic representation and accountability. In countries with weak policing legislatures, voters can expect parties in charge of a given policy to shape government policy output, which justifies electoral accountability along jurisdictional lines. Who shapes policy-making in coalition governments in countries with strong legislatures and thus whose preferences are represented and who should be held accountable at elections remain less clear. This further exacerbates the problem of blurred lines of responsibility in coalition settings. While previous research has shown that coalition partners can mitigate ministerial autonomy and move ministerial proposal closer to the coalition compromise (Martin & Vanberg, 2014), our results suggest that in prospect of legislative oversight, minister parties can strategically shelve policies, thus constraining the ability of coalition partners to make their desired policies part of the governmental policy output. When and which policies ministers are able to shelve, as well as which institutions can mitigate ministerial gatekeeping powers are central questions for policy-making and electoral accountability, which we hope future research will take upon. We also hope that our findings will stimulate scholars to divert their focus on the role of legislative institutions for electoral accountability in coalition settings.

## Supplemental Material

sj-pdf-1-cps-10.1177_00104140211024312 – Supplemental Material for Ministerial Autonomy, Parliamentary Scrutiny and Government Reform Output in Parliamentary Democracies Click here for additional data file. Supplemental Material, sj-pdf-1-cps-10.1177_00104140211024312 for Ministerial Autonomy, Parliamentary Scrutiny and Government Reform Output in Parliamentary Democracies by Hanna Bäck, Wolfgang C. Müller, Mariyana Angelova and Daniel Strobl in Comparative Political Studies

## Notes

1. Existing work on legislative policy-making reveals that parties in parliament structure policy-making in such a way to allow them to signal their true policy position and take credit for their attempt to serve the interests of their voters (see e.g. Martin & Vanberg, 2005, 2008, 2011).

2. They may be policy-seeking for instrumental or intrinsic reasons (see e.g. Müller & Strøm, 1999).

3. A recent study by Alexiadou and Hoepfner (2019) show that audience costs affect policy decisions in multiparty cabinets.

4. Replication materials and code can be found at (Bäck et al., 2021).

5. See Appendix A to C (Supplementary Material) for detailed information about the coding approach and Table A1 (Supplementary Material) for the policy categories and sub-categories.

6. We provide further information on the EIU and its reporting style and approach in Appendix A (Supplementary Material). We cross-validate our data by coding in addition more than 200 country reports issued annually or biannually by the OECD. From these country reports we extract additional information on the same reform measures, as well as add measures not mentioned in the EIU reports.

7. We provide intercoder reliability analyses accompanied with a detailed discussion of the result in Appendix C (Supplementary Material). We believe that the results substantiate the high quality of our data.

8. The database we used is available here: http://www.kolumbus.fi/taglarsson/dokumentit/governm2.htm.

9. We explain our approach and provide the full formula in Appendix D (Supplementary Material).

10. We run robustness checks with alternative measures, where we calculate the responsible minister alternation and coalition alternation by taking the absolute distance between the current and the *previous cabinet*. These alternation measures do not take into account the length of cabinet duration and make the strong assumption that ministers or the coalition as a whole in short-lived cabinets can influence the location of the policy status quo the same way as ministers or the coalition as a whole in long-lived cabinets do. We find weaker, but substantively comparable results when we use these alternation measures. The results are available upon request from the authors.

11. We are highly thankful to Lanny W. Martin and Georg Vanberg for providing their time-variant legislative policing strength index.

12. We present the predicted number of government reform measures for different levels of responsible minister alternation in legislatures with low, medium and high policing strength in Figure E1 in the Appendix (Supplementary Material).
