# Supplementary material for: Ministerial Autonomy, Parliamentary Scrutiny and Government Reform Output in Parliamentary Democracies
Source: Comp Polit Stud. 2021 Jul 2;55(2):254–86. doi: 10.1177/00104140211024312 (PMC8796161; doi:10.1177/00104140211024312)
Supplement: sj-pdf-1-cps-10.1177_00104140211024312 – Supplemental Material for Ministerial Autonomy, Parliamentary Scrutiny and Government Reform Output in Parliamentary Democracies [file sj-pdf-1-cps-10.1177_00104140211024312.pdf]

Appendix

A. Coding scheme and coding decisions

Our dataset comprises important individual reform measures, which were introduced and passed across European countries for a period of 20 years. For this purpose we coded more than 1000 country reports issued by the Economist Intelligence Unit (EIU). The EIU is a business unit of the Economist Group, which specializes in economic research, analysis and forecasting. It issues country reports on a regular basis, which contain information on the current policy situation and policy changes for countries across the globe. EIU's customers range from business investors, international organizations, and government agencies to academic institutes. The EIU's goal and target group ensure that the country reports include only important reforms that change the policy status quo and exclude incremental provisions. The EIU reports concrete policy changes and provides detailed information about individual reform measures included in a given law. A major advantage of the EIU reports is that they are prepared on a regular basis – quarterly, until 2007 and monthly, afterwards – which ensures a detailed and timely coverage.

EIU country reports are prepared by country experts with deep knowledge of policy developments in a given country. The EIU has over 70 years' experience in policy consulting and employs full-time more than 130 country specialists and analysts who speak 25 languages and operate globally. These country experts use various information channels, such as official statements, media reports and direct contact with government officials. According to interviews with former editors, country experts follow common general guidelines for their reporting. The whole process is supervised by the central editors to ensure comparability in the reporting procedure and structure across countries. We cross-validate our data by coding in addition more than 200 country reports issued annually or

biannually by the OECD. From these country reports we extract additional information on the same reform measures, as well as add measures not mentioned in the EIU reports.

We created a comprehensive codebook which allows us to code detailed information about the different stages, timing and content of important reform measures mentioned in the country reports. We code whether a reform is a reform ambition (planned reforms), an actual reform, or a failed reform. For the purposes of these analyses we focus only on actual reform measures and disregarded reform ambitions and failed reforms. We also code information about the different stages of a reform every time these are reported – the date when the reform was decided at the cabinet level, introduced into parliament, adopted and entered into force. Unfortunately, the EIU does not systematically provide information about each stage for each reform measure. While for a vast majority of cases we have information either about the adoption and the entrance into force dates, sometimes we only have information when the reform was decided at the cabinet level or initiated in parliament. Fortunately, the Economist Intelligence Unit also reports when reforms fail. We coded this information as well and excluded from our analyses all reform measures which were indicated as failed. Our dataset used in the analyses therefore should include only those measures which were introduced in parliament and subsequently adopted. While it is possible that the Economist Intelligence Unit did not report for some reform measures that they eventually failed, such instances should be exceptional – the Economist's country reports are market driven and any failure in reporting is costly for the investors who use this information. Therefore, for the reform measures for which the EIU did not report an adoption date, but also did not report as failed, we assume that these reform measures were adopted. Given the market incentives and the detailed reporting style of the Economist Intelligence Unit, we believe this is a reasonable and justified assumption.

For each reform we code the policy category it belongs to, as well as, whenever reported, accompanying information like the context of the reform measure, the reason for reform failure, conflict and disagreement about the reform from different parties, public opinion and

other meta information. We apply a comprehensive coding of the policy category where we differentiate between three levels: policy area (e.g. social policy, economy policy), policy category (e.g. product and market services, capital market, monetary policy, budget/public debt, old-age, disability, survivors, sickness and maternity, long-term care, unemployment, family, social welfare, general social security), and direction of the reform (e.g. price control, more liberalization/competition/deregulation, more regulation, subsidies increase, subsidies decrease, accepting international commitments, abolishing international commitments, insured's fees/contributions up, insured's fees/contributions down; employer contributions up, employer contributions down etc.) (for more information see Table A1).

We code individual reform measures, even when they are part of a given law or a law package. We code only those reform measures that specify the policy instrument used to introduce a policy change. We do not code vague phrases such as "the government has started to fight rising levels of unemployment" – this phrase does not indicate an actual policy instrument used to reduce unemployment levels. We code statements such as "In addition, a government pre-retirement scheme which takes older people off the Live Register has been extended to include those aged 55 to 58. The effect of these changes 'removed' 7,500 people from the May unemployment figures." (EIU 1992a, p. 19) Given our categories presented in Table A1, this reform measure is coded as social policy – old age, disability, survivors – increase number of beneficiaries/relax eligibility criteria. Below we list further examples of coded reform measures from the social and economic policy areas.

Table A1: Overview of coded policy areas, categories, sub-categories and direction of reform measures

	11 Categories*	28 Sub-Categories	Description of subcategory
ECONOMY	*1) Product Market and Services (1-8,15)*<br><br>*2) Capital Market (1-8,15)*<br><br>*3) Monetary Policy (9-11,15)*<br><br>*4) Budget/Public Debt (12-15)*	*1) Price control/adjustments;*	*Any regulations dealing with price control, setting limits, adjusting them*
		2) More liberalisation /competition / deregulation;	*Includes also consumer protection issues; regulations to stimulate capital market*
		3) More regulation;	*Includes all economic regulations except price controls; also includes consumer protection issues and credit regulations*
		4) Nationalization, establishment of new public companies /banks;	
		5) Privatization, less public control of companies;	
		6) Subsidies increase/new business development scheme;	*Subsidies to individual firms, to sectors, to individuals, credit subsidies; Incl. promotion of innovation*
		7) Subsidies decrease/ending of business development scheme;	
		8) Investments, investment incentives, business cycle policies.	*Includes public investments; new credit schemes*
		9) Adjust the goals/mandate or level of independence of the central bank;	
		10) Accepting international commitments (e.g. EMS);	
		11) Abolishing international commitments (e.g. EMS).	
		12) Current deficit policies;	*Includes general public savings and expenditure cuts*
		13) Institutional deficit rules;	*Such as the German Schuldenbremse*

		14) Debt management;	*All measures concerning the handling of existing debts, e.g. reimbursement, duration of government bonds*
		15) Other regulations and interventions	*Residual subcategory; can be coded with all categories*
SOCIAL	*5) Old-age, disability, survivors (16-28)*<br><br>*6) Sickness & maternity (16-28)*<br><br>*7) Long-term care (16-28)*<br><br>*8) Unemployment (16-28)*<br><br>*9) Family (16-28)*<br><br>*10) Social welfare (16-28)*<br><br>*11) General social security (16-28)*	*16) insured's fees/contributions up;*	*Includes insurance premia as well as payments and fees for certain social security issues*
		17) insured's fees/contributions down;	
		18) employer contributions up;	*Changes in the share that employers need to contribute to their workers' social insurances or services*
		19) employer contributions down;	
		20) state expenditure up;	*Changes in government spending on social security, e.g. government grants and payments for private insurance schemes*
		21) state expenditure down;	
		22) benefits/services up;	*Changes in the level of social benefits or the scope of services in the social security system, e.g. raising the number of child care places.*
		23) benefits/services down;	
		24) increase beneficiaries/relax eligibility criteria;	*Increase the number of people who are eligible to receive money (due to a change in eligibility criteria)*
		25) reduce beneficiaries / tighten eligibility criteria;	*Decrease the number of people who are eligible to receive money (due to a change in eligibility criteria)*
		26) undisclosed adjustments of contributions/benefits;	*Used as a default category for contributions and benefits if sub-categories 16-22 cannot be applied*
		27) limit benefit growth/system growth;	*Expenditure freeze*
		28) other (undisclosed changes in financing/organisation/new insurance/etc.)	*Includes e.g. organisational and administrative changes of a social insurance, introduction of new insurance.*

**Note: The corresponding sub-categories for each category are indicated in parentheses.*

"In December 1998 the government sold its remaining shareholding of 25.1% in CGER/ALSK to a Belgo-Dutch financial group (...). " (EIU 1999, p. 18) This sentence indicates a concrete action where the government sells it shareholdings. It was coded as a reform measure which came into force and was categorized as economic policy – product market and services - privatization.

"Mr. Reynolds has already forced the major oil companies to reduce petrol prices by 10p a gallon and diesel by 5p a gallon with the Introduction of Maximum Price Orders." (EIU 1987, p. 14) Here, we find action of price adjustment and this reform measure was coded as economic policy – product market and services – price control/adjustment.

One sentence can contain more than one measure.

"An incomes policy was finally agreed at the end of November and the 1992 budget was eventually approved by the Eduskunta.

[...]

As of 1993, <u>wage earners will start to pay a 3 per cent compulsory earnings related pension contribution</u>, of which 2.5 per cent will be used to reduce employers' contributions." (EIU 1992b, p. 10). In this example we coded two reform measures. The underlined part was coded as an increase in social insurance fees and contributions for the insured. In particular as social policy – old-age/disability/survivors – insured's fees/contributions up. The remaining part of the sentence was coded as a decrease in employer contributions, in particular, as social policy – old-age/disability/survivors – employer contribution down.

One reform measure can span over several sentences.

> *"On July 5th the government's pension and health reforms were adopted by parliament in the face of strong opposition.*
>
> *[...]*

Pension contributions by civil servants will increase by 0.8 percentage points (from 11.75% to 12.55% of income), and for the self-employed and farmers by 0.5 percentage points (from 14.5% to 15% of income). However, for the self-employed health insurance contributions will be reduced by 0.2 percentage points, thus leaving a net increase of only 0.3 percentage points." (EIU 2000, p. 18) Here we coded two reform measure. The first measure refers to an increase of insured's pension contributions and was coded as social policy – old age, disability, survivors – *insured's fees/contributions up*. The second reform measure (underlined) decreases the health insurance contributions of the insured ones and was coded as social policy – sickness and maternity – *insured's fees/contributions down.*

EIU reports indicate a reform measure not only when a reform is passed or enters into force, but also when it is discussed at the cabinet level or initiated in parliament. Therefore, sometimes the different stages of a reform measure are reported over several country reports. For instance:

"On June 16th the government's pension reform plan (réforme des retraites) was unveiled by the labour, solidarity and civil service minister, Mr Woerth, and the junior minister responsible for the civil service, Georges Tron.

[...]

On June 24th, French trade unions organised widespread strike action in protest.

[...]

The government considered various means of reducing the financial imbalances projected for the French pension system over the long term (May 2010, Economic policy), but in the end decided to raise the statutory age of retirement from 60 years to 62 years." (EIU 2010a, p. 16–17)

“The government's reform of the pension system was finally enacted in mid- November, following a wave of strikes and public protests.” (France, EIU November 2010b, p. 5)

This example presents a pension reform that increases the retirement age from 60 years to 62 years and was enacted in France in 2010. This reform measure was coded as social policy – old-age/disability/survivors – reduce beneficiaries/tighten eligibility criteria.

B. Coder training and coding process

Coders are trained student research assistants who study political science or economics and have excellent English skills. Every coder undergoes a training period of about one month. In the first weeks, coders take part in training sessions where each coding category is discussed in detail. In addition, coders do practical exercises where they go through a country report together with the project researcher. The following weeks coders code several country reports to get aquatint with the format and style of the country reports as well as to learn how to code. Student coders receive constant feedback during the training period.

The actual coding starts after the training period. Student coders first read the country report, highlight and annotate the parts that need to be coded and then transfer their coding decisions to a user-friendly access database. The coding process is supervised by the project researcher, where especially in the beginning coders receive regular and frequent feedback on their coding. In particular, at the early stages of the coding, feedback is given on every handful of reports. Student coding is supervised and modified if needed by the project researcher. In particular, the project researcher reads the same reports by him- or herself and marks the places that need to be coded. Then the researcher opens the database with the student's coding and checks the correctness of the coding – what has been coded and how. Therefore, every country report and coding is practically coded by two people – the student coder and the project researcher. This approach reduces coding error and ensures higher quality of the data.

C. Reliability analysis

In addition to the de facto double coding by student coders and project researchers we have conducted classical reliability checks among student coders, which we report below.

Coding of Netherlands and France

Two student assistants were assigned to code six EIU country reports for Netherlands covering 1981–1983. For The Netherlands students coders coded EIU country reports from October 1981, January 1982, April 1982, June 1982, October 1982 and January 1983. Another two student assistants coded 15 EIU country reports for France, covering 2007 and 2008. For France a student coder coded EIU country reports from July 2007, October 2007, November 2007, December 2007, January 2008, February 2008, March 2008, April 2008, May 2008, June 2008, July 2008, August 2008, September 2008, October 2008, and November 2008.[1] *All four coders were newly trained student assistants. They were asked to read the coding scheme, participated in two workshops where the coding scheme was discussed in detail, and coded several country reports as part of their training. All student coders were assigned the double coding after several months of coding practice.*

Main results

The following analysis presents reliability analyses for Netherlands and France. We report our double coding of actual reform measures.[2] *We calculate three measures of inter-coder reliability. To test unitizing reliability, a commonly reported measure is* ***Guetzkow's*** **U** *(Guetzkow 1950), which shows the disagreement between the number of reforms coded by*

[1] The difference in the number of EIU reports is a result of the fact that in 2007 EIU switched from quarterly to monthly reports.

[2] *Our dataset also covers reform ambitions and failed reforms, which are not considered in the analyses of this paper.*

coder A with the number of reforms coded by coder B: $Guetzkow's\ U = \frac{\text{sum of codings by coder A}-\text{sum of codings by coder B}}{\text{sum of codings by coder A}+\text{sum of codings by coder B}}$.

Values below 0.1 (less than 10% disagreement) are generally regarded as a good benchmark. Given that our dependent variable in the main analysis is the number of reforms, the disagreement measure is particularly useful for intercoder reliability in our case.

However, the disagreement measure does not reveal whether coders code the same measures. We therefore also calculate the **index of coterminability (ioc)** *as defined by Angelmar and Stern (1978)* $ioc = \frac{\text{number of records with coder agreement}}{(\text{records with agreement}+\text{records without agreement})}$.

This agreement measure shows the agreement between the measures contained in the two datasets produced by both coders. This measure ranges between 0 to 1, where 1 indicates perfect agreement between the two coders, in other words that both coders coded the same measures and no reform measure was coded only by one of the coders.

Note that the disagreement and the agreement measures are scores based on percentages, which do not take into account agreement by chance (e.g. by Krippendorff 2004). However, given that the EIU reports are 20 pages long on average and the coders do not code every sentence, but only those sections that contain a reform measure, it is highly unlikely that they will arrive by chance at the same units from the text.

Nevertheless, we also calculate **Krippendoffs'a Alpha** *coefficient for all matched cases, which reveals the inter-coder agreement for the policy categorization of each reform. The codes used in this paper are from two policy levels: 1)* **policy area**, *where coders can choose between 4 different areas, and 2)* **policy category**, *where coders can choose between 20 categories).*

Table C1: Actual Reform Measures

**Test**	**Result**
Disagreement	*0.0552*
Index of Coterminability (IOC)	*0.6864*
Krippendorff's alpha (4 policy area)<br>*2 raters, 81 cases*	*0.8344*
Krippendorff's alpha (20 policy categories)<br>*2 raters, 81 cases*	*0.7837*

Table C1 presents the results of the double coding of actual reform measures mentioned in EIU country reports. Our intercoder analyses show about 6% disagreement in the number of coded reform measures and hence 94% agreement in the number of coded reform measures. It is possible that while the number of reform measures coded by two coders is the same, that they code different measures. We therefore calculate an index of coterminability (IOC), which shows the proportion of the same coded measures. Our analyses reveal a coterminability coefficient of about 0.69.[3] *Furthermore, we also calculate Krippendorff's Alpha coefficient, which reveals the inter-coder agreement for the policy categorization of the reform measures. We report a Krippendorff's Alpha coefficient of 0.83 for the policy area level, which is used in this paper. We find a Krippendorff's Alpha coefficient of 0.78 for the policy categories level, which encompasses 20 policy categories.*

One of the best features of our reform data is its disaggregated level, where instead of focusing on laws which can include several reform measures and package deals, our data considers the lowest possible unit of analyses – reform measure. Accordingly, even if several reform measures were part of one law or reform package these are coded separately in our dataset. However, even this disaggregated level can be further subdivided, for example in

[3] *If we consider only coding of the social and economic policy areas, then the disagreement coefficient is 0.0491 and the index of coterminabilty is 0.7183.*

cases like reform measures which increase social security benefits for different groups – e.g. low and high income earners. Such cases inevitably increase the level of interpretation. We therefore identified such instances in the database and aggregated them. We did the same for our sample of double coding. Below we present the reliability checks results after this aggregation (Table C2). Indeed, the analyses yield slightly better results, however these are substantively identical to the original analyses. In particular, we find 4% disagreement (hence 96% agreement) in the number of coded reform measures, 0.70 index of coterminability[4] and 0.83 Krippendorff's alpha for the policy level.

Table C2: Actual reforms, aggregated.

Test	*Result*
Disagreement	*0.0416*
Index of Coterminability (IOC)	*0.6991*
Krippendorff's alpha (policy area) (4)	*0.8310*
Krippendorff's alpha (policy categories) (20)	*0.7801*

[4] *If we consider only coding of the social and economic policy areas then the disagreement coefficient is 0.0344 and then the index of coterminabilty is 0.7313.*

D. Policy positions and saliency

MARPOR categories for socioeconomic left–right position of parties

Table D1 shows the 14 MARPOR categories used to construct the socio-economic left–right placements of responsible parties on a one-dimensional left–right scale (Volkens et al. 2015). We follow the method for scaling continuous left–right positions from political text suggested by Lowe et al (2011a, b). In particular, we use the 14 categories and calculate a logged left–right ratio following the formula in Lowe et al. (2011a: 131).

Table D1: MARPOR categories used for socioeconomic left–right dimension

Left		*Right*	
per403	*Market Regulation: Positive*	*per401*	*Free Market Economy*
per404	*Economic Planning: Positive*	*per402*	*Incentives: Positive*
per406	*Protectionism: Positive*	*per407*	*Protectionism: Negative*
per412	*Controlled Economy: Positive*	*per414*	*Economic Orthodoxy*
per413	*Nationalisation: Positive*	*per505*	*Welfare State Limitation*
per503	*Equality: Positive*	*per702*	*Labour Groups: Negative*
per504	*Welfare State Expansion: Positive*		
per701	*Labour Groups: Positive*		

Left–right policy positions were calculated following Lowe et al. (2011a) and the accompanying Codebook, equation 9 (Lowe et al. 2011b, p. 2):

Left–right policy position = $\log(\mathrm{R}^{\mathrm{PER}} + 50/N) - \log(\mathrm{L}^{\mathrm{PER}} + 50/N)$

Importance scales were calculated following Lowe et al. (2011a) and the accompanying Codebook (Lowe et al. 2011b, p. 3):

Saliency measure $= \log(\mathrm{R}^{\mathrm{PER}} + \mathrm{L}^{\mathrm{PER}} + 100/N) - log100$

$\mathrm{R}^{\mathrm{PER}}$ ($\mathrm{L}^{\mathrm{PER}}$) *are equal to the sum of all Right PER (Left PER) categories listed in Table A2.*

To calculate the seat and saliency-weighted coalition compromise position, we follow Martin and Vanberg (2014). We first calculate party seats saliency weights by multiplying the issue saliency for a party (as a proportion of the sum of the saliences of all government parties) with the party's cabinet seat share. We then normalize this product so that the products of all government parties sum to 1. The coalition compromise position is then calculated by multiplying a government party's policy position with the corresponding normalized party seats saliency weight and taking the sum of the weighted positions of all government parties.

More specifically, the seat and saliency weighted coalition compromise positions is calculated as such:

$$\frac{\left(P_{party\,1} \times \frac{I_{party\,1}}{I_{party\,1} + \cdots + I_{party\,n}} \times \frac{S_{paty\,1}}{S_{party\,1} + \cdots + S_{party\,n}}\right) + \cdots + \left(P_{party\,n} \times \frac{I_{party\,n}}{I_{party\,1} + \cdots + I_{party\,n}} \times \frac{S_{paty\,n}}{S_{party\,1} + \cdots + S_{party\,n}}\right)}{\left(\frac{I_{party\,1}}{I_{party\,1} + \cdots + I_{party\,n}} \times \frac{S_{paty\,1}}{S_{party\,1} + \cdots + S_{party\,n}}\right) + \cdots + \left(\frac{I_{party\,n}}{I_{party\,1} + I_{party\,2} + \cdots + I_{party\,n}} \times \frac{S_{paty\,n}}{S_{party\,1} + \cdots + S_{party\,n}}\right)}$$

where n stands for a government party (party 1, 2, 3 etc.), P for the corresponding party position, I for the corresponding policy saliency (importance) and S for the corresponding legislative seats.

After multiplying each term by the lowest common denominator $\left(I_{party\,1} + \cdots + I_{party\,n}\right) \times \left(S_{party\,1} + \cdots + S_{party\,n}\right)$ *the above equation simplifies to*

$$\frac{\left(P_{party\ 1}\ \times\ I_{party\ 1}\ \times\ S_{party\ 1}\right) + \cdots +\ (P_{party\ n}\ \times\ I_{party\ n}\ \times\ S_{party\ n})}{(I_{party\ 1} \times\ S_{party\ 1}) + \cdots + (I_{party\ n} \times\ S_{party\ n})}$$

Using different annotation this is equivalent to $\sum_{1}^{n} \frac{P_n \times I_n\ \times S_n}{\sum_{1}^{n}(I_n \times S_n)}$

E. Further substantive effects from the main models and robustness analyses

We present further substantive effects from model 2 (see regression results in Table 2 in the paper) in Figure E1, where we show the predicted number of government reform measures for different levels of responsible minister alternation in legislatures with low, medium and high policing strength. In weak legislatures with policing strength of about -1.9 (e.g. Ireland in the 1980s and early 1990s) (see top left panel in Figure E1), higher ideological difference between the current and the previous responsible minister party(ies) results in a higher number of government reform measures.

Figure E1: Expected Number of Government Social and Economic Policy Reform Measures for Different Levels of Responsible Minister Alternation and Legislative Policing Strength

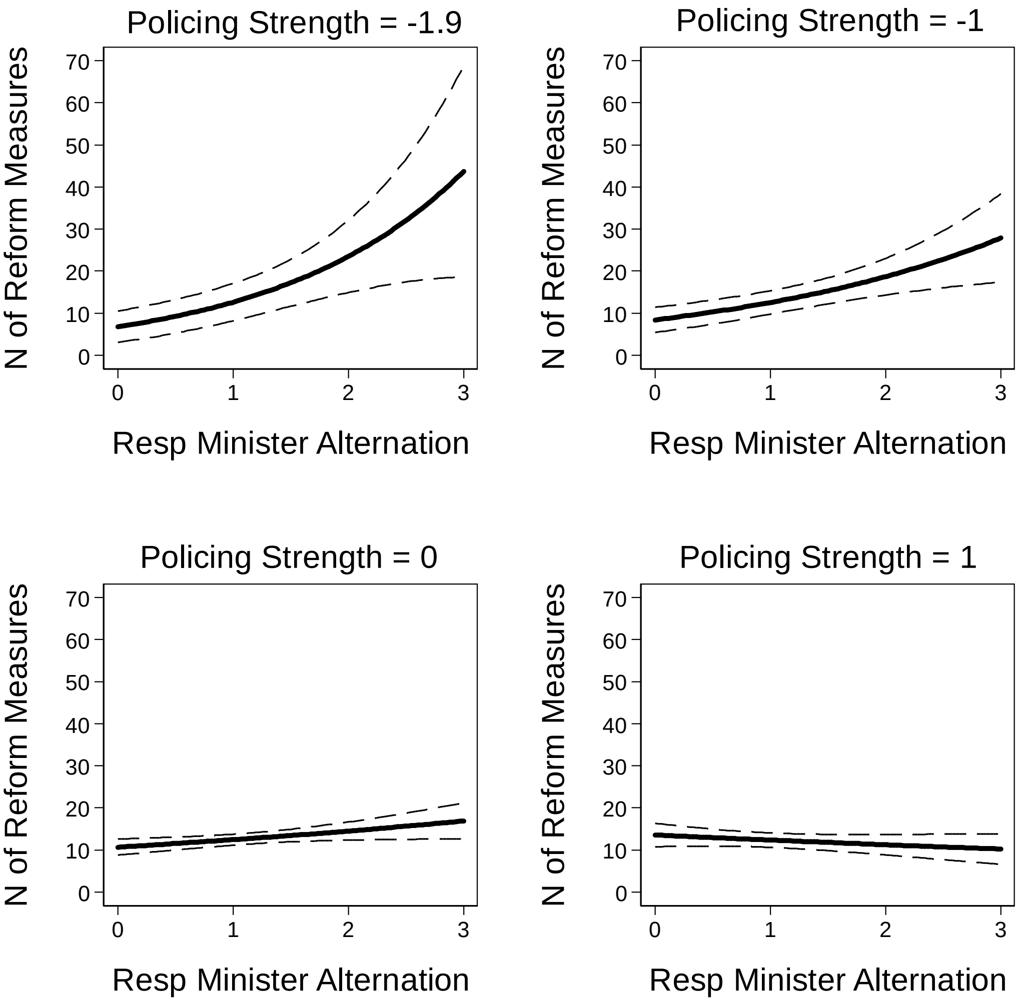

Note: *Figure E1 is based on Model 2, Table 2, with 95% confidence bounds. Responsible minister alternation ranges from 0 to 95th percentile of its distribution for presentation reasons.*

For example, 0 ideological discrepancy between the current and previous responsible minister party results in about 7 reform measures, while very high responsible minister alternation (e.g. 3) results in about 43 reform measures. At the same time we see that in strong legislatures (e.g. Germany, Netherlands or Austria with policing strength values of about 1), governments reform productivity is independent from responsible minister alternation – low as well as high responsible minister alternation results in about the same number of reform measures (see bottom right panel in Figure E1).

Model A1 (see Table E1 in the Appendix) runs a multilevel mixed-effects negative binomial model where both, the responsible minister alternation and coalition alternation, are interacted with the legislative policing index. We include the same control variables as our main models from the paper. We find substantively equivalent results to the models 2 and 3 from the main text, where only one of the alternation variables is interacted with the legislative policing index. As the results in Table E1 show, both interactions are negative. However, the responsible minister interaction is significant only at the 0.1 level and the coalition alternation interaction is not significant anymore. Overall, these results are in line with our main findings, they also show that responsible minister alternation and coalition alternation effect declines with higher legislative policing powers.

The larger confidence intervals and lower significance levels might be due to the higher correlation between the covariates because of the additional interaction terms. This is revealed in the correlation matrix (see Table E2) and multicollinearity diagnostics for this model (see Table E3). We can see rather high correlation between the two interaction terms (0.83). In addition, we also find that the variance inflation factor (VIF) is above 5 for the two interaction terms (see Table E3), which following a conservative threshold indicates high multicollinearity (Sheather 2009). In contrast, the variance inflation factor is below 5 for all variables and interaction terms included in our main models 2 and 3 from the main text. See the multicollinearity diagnostics for Model 2 and Model 3 presented in Tables E4 and E5 respectively.

Table E1: Multilevel Mixed-Effects Negative Binomial Model of the Number of Social and Economic Government Reform Measures in Coalition Governments (Additional Models)

	Model A1
Responsible Minister Alternation (RMA)	*1.12*
	(0.09)
Coalition Alternation (CA)	*0.89*
	(0.07)
Legislative Policing Index	*1.24~*
	(0.15)
*RMA * Legislative Policing Index*	*0.84~*
	(0.09)
*CA * Legislative Policing Index*	*0.91*
	(0.10)
Number of coalition parties	*1.03*
	(0.03)
Responsible Minister – Coalition Distance	*1.16~*
	(0.10)
Average Δ in GDP growth	*0.92****
	(0.02)
Unemployment Crisis (dummy)	*1.16*
	(0.19)
Average Δ in Inflation Rate	*0.96*
	(0.03)
Cabinet Duration (years)	*1.61****
	(0.05)
Social (vs. economic) policy	*1.00*
	(0.07)
ln(alpha)	*0.09****
	(0.02)
Random intercept (Country level)	*1.01*
	(0.01)
Observations	*150*
Log likelihood	*-443*
AIC	*915*

Table E2: Correlation Matrix

	N reforms (DV)	RMA	CA	Legislative Policing Index	RM-Coalition Distance	N cabinet parties	Average Δin GDP growth	Unemploymen t Crisis (dummy)	Average Δin Inflation Rate	Cabinet Duration (years)	Policy	RMA*Policin g Index	CA*Policing Index ex
N reforms (DV)	1.00												
RMA	0.18*	1.00											
CA	0.17*	0.66***	1.00										
Legislative Policing Index	0.04	-0.14	0.05	1.00									
RM-Coalition Distance	0.04	0.10	-0.03	0.10	1.00								
N cabinet parties	-0.10	-0.07	-0.16*	0.18*	-0.11	1.00							
Average Δin GDP growth	0.03	0.09	0.01	-0.42***	0.10	-0.03	1.00						
Unemployment Crisis (dummy)	-0.00	-0.06	-0.21**	-0.40***	0.12	-0.07	0.14	1.00					
Average Δin Inflation Rate	-0.18*	-0.13	-0.09	-0.01	-0.09	0.23**	-0.12	-0.11	1.00				
Cabinet Duration (years)	0.72***	0.20*	0.26**	0.03	-0.02	-0.17*	0.28***	-0.06	-0.09	1.00			
Policy	0.00	0.04	-0.01	0.01	-0.16	0.00	-0.01	-0.00	-0.00	-0.00	1.00		
RMA*Policing Index	-0.09	-0.06	-0.03	0.78***	0.05	0.14	-0.31***	-0.15	0.02	0.03	0.02	1.00	
CA*Policing Index	-0.09	-0.18*	0.06	0.63***	0.13	0.05	-0.11	-0.10	-0.01	0.12	0.01	0.83***	1.00

$^{*}\ p < 0.05$, $^{**}\ p < 0.01$, $^{***}\ p < 0.001$

Table E3: Collinearity Diagnostics for Model A1 from Table E1

Variable	*VIF*	*SQRT VIF*	*Tolerance*	*R-Squared*
RMA	*3.10*	*1.76*	*0.3222*	*0.6778*
CA	*2.86*	*1.69*	*0.35*	*0.65*
Legislative Policing Index	*4.67*	*2.16*	*0.2140*	*0.7860*
RM-Coalition Distance	*1.34*	*1.16*	*0.7479*	*0.2521*
*RMA*policing index*	*8.40*	*2.90*	*0.1190*	*0.8810*
*CA*policing index*	*5.47*	*2.34*	*0.1828*	*0.8172*
Number of cabinet parties	*1.23*	*1.11*	*0.8128*	*0.1872*
Average Δ in GDP growth	*1.58*	*1.26*	*0.6326*	*0.3674*
Unemployment Crisis (dummy)	*1.47*	*1.21*	*0.6824*	*0.3176*
Average Δ in Inflation Rate	*1.14*	*1.07*	*0.8796*	*0.1204*
Cabinet duration	*1.27*	*1.13*	*0.7901*	*0.2099*
policy	*1.04*	*1.02*	*0.9581*	*0.0419*

Note: Mean VIF 2.80

Table E4: Collinearity Diagnostics for Model 2 (Table 2)

Variable	*VIF*	*SQRT VIF*	*Tolerance*	*R-Squared*
RMA	*2.15*	*1.47*	*0.4641*	*0.5359*
CA	*2.16*	*1.47*	*0.4633*	*0.5367*
Legislative Policing Index	*4.50*	*2.12*	*0.2221*	*0.7779*
RM-Coalition Distance	*1.22*	*1.11*	*0.8170*	*0.1830*
*RMA*policing index*	*3.05*	*1.75*	*0.3281*	*0.6719*
Number of cabinet parties	*1.23*	*1.11*	*0.8130*	*0.1870*
Average Δ in GDP growth	*1.48*	*1.22*	*0.6760*	*0.3240*
Unemployment Crisis (dummy)	*1.46*	*1.21*	*0.6828*	*0.3172*
Average Δ in Inflation Rate	*1.14*	*1.07*	*0.8808*	*0.1192*
Cabinet duration	*1.26*	*1.12*	*0.7947*	*0.2053*
policy	*1.04*	*1.02*	*0.9627*	*0.0373*

Note: Mean VIF 1.88

Table E5: Collinearity Diagnostics for Model 3 (Table 2)

Variable	*VIF*	*SQRT VIF*	*Tolerance*	*R-Squared*
RMA	*2.12*	*1.46*	*0.4707*	*0.5293*
CA	*2.14*	*1.46*	*0.4665*	*0.5335*
Legislative Policing Index	*2.92*	*1.71*	*0.3428*	*0.6572*
RM-Coalition Distance	*1.20*	*1.09*	*0.8355*	*0.1645*
*CA*policing index*	*1.99*	*1.41*	*0.5037*	*0.4963*
Number of cabinet parties	*1.23*	*1.11*	*0.8135*	*0.1865*
Average Δ in GDP growth	*1.54*	*1.24*	*0.6483*	*0.3517*
Unemployment Crisis (dummy)	*1.40*	*1.18*	*0.7131*	*0.2869*
Average Δ in Inflation Rate	*1.13*	*1.06*	*0.8878*	*0.1122*
Cabinet duration	*1.26*	*1.12*	*0.7924*	*0.2076*
policy	*1.04*	*1.02*	*0.9622*	*0.0378*

Mean VIF 1.63

Fixed Effects Models

We run negative binomial models with fixed effects by country and policy area and clustered standard errors by country with the same variables as in Model 1, 2, and 3 from the main text (see Table 2) and Model A1 from the Appendix (see Table E1). We present our results in Table E6. Both, the multilevel models from the main text and the fixed effects models yield substantively identical results.

Table E6: Negative Binomial Models of the Number of Social and Economic Government Reform Measures in Coalition Governments (Fixed Effects Models)

	Model FE1	*Model FE2*	*Model FE3*	*Model FE4*
Responsible Minister Alternation (RMA)	*1.12*	*1.18****	*1.08*	*1.14**
	(0.08)	*(0.05)*	*(0.06)*	*(0.07)*
Coalition Alternation (CA)	*0.93*	*0.87~*	*0.94*	*0.90*
	(0.06)	*(0.06)*	*(0.06)*	*(0.06)*
Legislative Policing Index		*0.98*	*0.77*	*0.90*
		(0.18)	*(0.13)*	*(0.12)*
RMA # Legislative Policing Index		*0.82****		*0.87~*
		(0.04)		*(0.07)*
CA # Legislative Policing Index			*0.82****	*0.92*
			(0.05)	*(0.10)*
Number of coalition parties	*0.96*	*0.97*	*0.97*	*0.97*
	(0.05)	*(0.04)*	*(0.05)*	*(0.04)*
Responsible Minister – Coalition Distance	*1.19~*	*1.14*	*1.20~*	*1.16*
	(0.12)	*(0.11)*	*(0.13)*	*(0.12)*
Average Δ in GDP growth	*0.94***	*0.93****	*0.94****	*0.93****
	(0.02)	*(0.02)*	*(0.02)*	*(0.02)*
Unemployment Crisis (dummy)	*1.24~*	*1.24~*	*1.16*	*1.21*
	(0.15)	*(0.15)*	*(0.15)*	*(0.14)*
Average Δ in Inflation Rate	*0.94*	*0.94*	*0.93*	*0.93*
	(0.05)	*(0.05)*	*(0.05)*	*(0.05)*
Cabinet Duration (years)	*1.63****	*1.66****	*1.67****	*1.66****
	(0.07)	*(0.07)*	*(0.07)*	*(0.07)*
Social (vs. economic) policy	*1.01*	*1.00*	*1.01*	*1.00*
	(0.08)	*(0.08)*	*(0.08)*	*(0.08)*
Belgium	*0.93*	*0.81*	*0.77~*	*0.78*
	(0.13)	*(0.14)*	*(0.11)*	*(0.13)*
Denmark	*0.70****	*0.75****	*0.75****	*0.76****
	(0.05)	*(0.05)*	*(0.06)*	*(0.06)*
Finland	*0.88*	*0.79*	*0.77~*	*0.77~*
	(0.12)	*(0.12)*	*(0.11)*	*(0.11)*
France	*1.27**	*0.65*	*0.51~*	*0.56*
	(0.14)	*(0.33)*	*(0.18)*	*(0.21)*
Germany	*0.88*	*0.83**	*0.89*	*0.85*
	(0.07)	*(0.08)*	*(0.09)*	*(0.08)*
Ireland	*0.73**	*0.45*	*0.34**	*0.39**
	(0.10)	*(0.23)*	*(0.15)*	*(0.17)*
Italy	*1.55**	*1.34*	*1.32*	*1.31*
	(0.35)	*(0.27)*	*(0.27)*	*(0.26)*
Netherlands	*0.86**	*0.78**	*0.76**	*0.77**
	(0.06)	*(0.10)*	*(0.08)*	*(0.09)*
Lnalpha	*0.09****	*0.08****	*0.09****	*0.08****
	(0.02)	*(0.01)*	*(0.02)*	*(0.02)*

Observations	*150*	*150*	*150*	*150*
Ll	*-439*	*-435*	*-435*	*-435*
Aic	*893*	*886*	*887*	*885*

Exponentiated coefficients; Standard errors in parentheses. $^{\sim}$ $p < .1$, * $p < .05$, ** $p < .01$, *** $p < 0.001$

Legislative policing index

Our analyses rely on the time variant legislative policing index provided by Martin and Vanberg (2020). Their original measure varies by country and year. Since our unit of analyses is cabinet, we calculated a time weighted average of the legislative policing index for each cabinet and country. We present the distribution of this variable in Figure E2. Each point indicates the year of cabinet begin in a given country. Note that our analyses includes only coalition cabinets within the rough time frame 1985-2005 and therefore does not include all cabinets depicted in the Figure E2.

Figure E2: Legislative Policing Strength Index – Cabinet Average Values

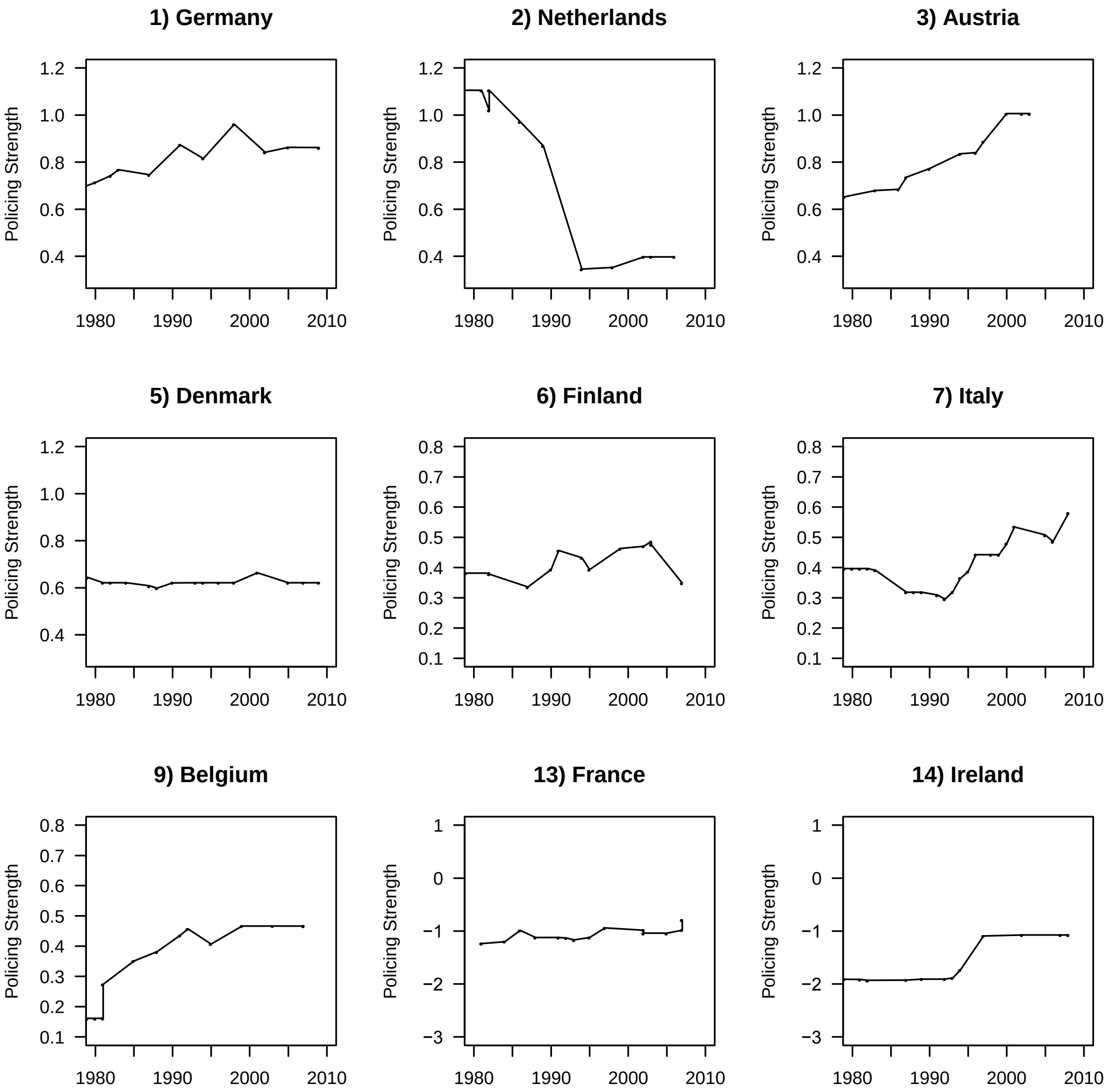

Note: The figure presents average policing strength scores by cabinet over time. Country rankings from 1 to 15 are depicted before the country name and are based on country averages (Martin and Vanberg 2020 and Figure 5). Note that the policing strength scales on the y-axis differ by strong legislatures (Germany, Netherlands, Austria, Denmark), moderate legislatures (Finland, Italy, Belgium) and weak legislatures (France and Ireland).

References

Angelmar, R. and L. W. Stern (1978). Development of a Content Analytic System for Analysis of Bargaining Communication in Marketing. Journal of Marketing Research, 15(1): 93-102.

Guetzkow, H. (1950). Unitizing and categorizing problems in coding qualitative data. Journal of Clinical Psychology, 6: 47–58.

EIU (1987) Ireland. Country report. No.3-1987. London: Economist Intelligence Unit.

EIU (1992a) Ireland. Country report. No.3-1992. London: Economist Intelligence Unit.

EIU (1992b) Finland. Country report. No.1-1992. London: Economist Intelligence Unit.

EIU (1999) Belgium Luxembourg. Country report 1st quarter 1999. London: Economist Intelligence Unit.

EIU (2000) Austria. Country report. August 2000. London: Economist Intelligence Unit.

EIU (2010a) France. Country report. July 2010. London: Economist Intelligence Unit.

EIU (2010b) France. Country report. November 2010. London: Economist Intelligence Unit.

Krippendorff, K. (2004). Reliability in Content Analysis: Some Common Misconceptions and Recommendations. Human Communication Research, 30(3): 411–433.

Lowe, W., Benoit, K., Mikhaylov, S., and Laver, M. (2011a). Scaling policy preferences from coded political texts. Legislative Studies Quarterly, 36(1): 123–155.

Lowe, W., Benoit, K., Mikhaylov, S., and Laver, M. (2011b). "Codebook.pdf", Replication data for: The Manifesto Project data extended to include the logit scales and standard. Available at: https://dataverse.harvard.edu/dataset.xhtml?persistentId=hdl:1902.1/17073 (last accessed 8 November 2020)

Martin, L. W., and Vanberg, G. (2014). Parties and policymaking in multiparty governments: The legislative median, ministerial autonomy, and the coalition compromise. American Journal of Political Science, 58(4): 979–996.

Martin, L., and Vanberg, G. (2020). Coalition governance, legislative institutions, and government policy in parliamentary democracies. American Journal of Political Science, 64(2): 325–340.

Sheather, S. (2009). A Modern Approach to Regression with R. New York: Springer Science + Business Media.

Volkens, A., Lehmann, P., Merz, N., Regel, S., and Werner, A. (2015). The manifesto data collection. Manifesto Project (MRG/CMP/MARPOR). Version 2015a. Berlin: Wissenschaftszentrum Berlin für Sozialforschung (WZB).
